# The ABC transporter A7 modulates neuroinflammation *via* NLRP3 inflammasome in Alzheimer’s disease mice

**DOI:** 10.1186/s13195-025-01673-2

**Published:** 2025-01-27

**Authors:** Irene Santos-García, Pablo Bascuñana, Mirjam Brackhan, María Villa, Ivan Eiriz, Thomas Brüning, Jens Pahnke

**Affiliations:** 1 Translational Neurodegeneration Research and Neuropathology Lab, Department of Clinical Medicine (KlinMed), Medical Faculty, University of Oslo (UiO) and Section of Neuropathology Research, Department of Pathology (PAT), Clinics for Laboratory Medicine (KLM), Oslo University Hospital (OUS), Sognsvannsveien 20, Oslo, NO-0372 Norway; 2 Institute of Nutritional Medicine (INUM), Lübeck Institute of Dermatology (LIED), University of Lübeck (UzL) and University Medical Center Schleswig-Holstein (UKSH), Ratzeburger Allee 160, DE-23538 Lübeck, Germany; 3 Department of Neuromedicine and Neuroscience, The Faculty of Medicine and Life Sciences, University of Latvia (LU), Jelgavas iela 3, Rīga, LV-1004 Latvia; 4 Department of Neurobiology, School of Neurobiology, Biochemistry and Biophysics, The Georg S. Wise Faculty of Life Sciences, Tel Aviv University (TAU), Ramat Aviv, IL-6997801 Israel; 5 Brain Mapping Group, Hospital Clínico San Carlos, IdISSC, Madrid, Spain

**Keywords:** Alzheimer’s disease, ABCA7, Microglia, Astrocytes, NLRP3, Inflammasome, APPPS1-21, Cx3cr1, ABC transporter

## Abstract

**Background:**

Specific genetic variants in the ATP-binding cassette transporter A7 locus (*ABCA7*) are associated with an increased risk of Alzheimer’s disease (AD). ABCA7 transports lipids from/across cell membranes, regulates Aβ peptide processing and clearance, and modulates microglial and T-cell functions to maintain immune homeostasis in the brain. During AD pathogenesis, neuroinflammation is one of the key mechanisms involved. Therefore, we wanted to investigate the specific role of ABCA7 in microglial activation via the NLRP3 inflammasome.

**Methods:**

We developed the first humanized, Cre-inducible *ABCA7*^*flx*^ knock-in mouse model, crossbred it with the APPPS1-21 β-amyloidosis model, and generated constitutive ABCA7^ko^ and microglia *Cx3cr1*-specific conditional ABCA7^ko^ AD mice. The role of ABCA7 was analyzed using histological, biochemical, molecular and mass spectrometry methods.

**Results:**

Constitutive knockout of the *Abca7* gene in APPPS1 mice increased the levels of Aβ42 and the number of IBA1+ (microglia) and GFAP+ (astrocytes) cells. Changes in the levels of astrocytes and microglia are associated with the activation of the NLRP3 inflammasome and increased levels of proinflammatory cytokines, such as IL1β and TNFα. Interestingly, microglia-specific ABCA7^ko^ restored Aβ_42_ peptide levels and IBA1^+^ and GFAP^+^ and NLRP3-related gene expression to the original APPPS1 mouse levels. In primary glial cell cultures of APPPS1-hA7^ko^ microglia and APPPS1 astrocytes from newborn pups, we observed that conditioned media from LPS-stimulated microglia was able to induce NLRP3 inflammasome expression and proinflammatory cytokine release in astrocytes.

**Conclusions:**

Our data suggest that ABCA7 transporters regulate the communication between microglia and astrocytes through the NLRP3 inflammasome and the release of proinflammatory cytokines. This regulation implicates ABCA7 as a key driver ultimately involved in the persistence of the inflammatory response observed in AD.

**Supplementary Information:**

The online version contains supplementary material available at 10.1186/s13195-025-01673-2.

## Introduction

Alzheimer’s disease (AD) is the most common, age-related neurodegenerative disorder, accounting for 60–80% of all dementias and affecting approximately 47 million people worldwide [1]. The majority of AD patients have a sporadic form of the disease (late-onset AD, LOAD), while almost 1% have a familial early-onset form (FAD) with Mendelian inheritance (< 60 years). However, both forms of AD are characterized by the accumulation of amyloid-β (Aβ) peptides and hyperphosphorylated tau protein, both of which cause neuronal loss, mainly in the hippocampus and cortex, leading to cognitive decline with memory loss and behavioral problems [2].

In 2011, a genome-wide association study (GWAS) identified *ABCA7* as a genetic risk factor locus for sporadic LOAD, which is now regarded as the second most important risk locus [3]. The *ABCA7* gene encodes the ATP-binding cassette (ABC) transporter A7, which is involved in the transport of lipids across cell membranes, including cholesterol and phospholipids [4], and the regulation of amyloid precursor protein (APP) processing to produce Aβ peptides [5]. Patients with *ABCA7* single nucleotide polymorphisms (SNPs) are more likely to develop AD, suggesting that a functional ABCA7 transporter is crucial for protecting against AD onset, possibly through its interaction with cell membrane lipids and mediation of cholesterol transport [6]. Notably, accumulating evidence suggests that ABCA7 also plays a critical role in regulating immune responses [7]. ABCA7 deficiency reduces the phagocytic clearance of Aβ in macrophages and microglia [8–10] and impairs cytokine responses in natural killer T cells [11].

Within the central nervous system (CNS), microglia and astrocytes are vital components of the nervous system. The bidirectional communication between neuroinflammation, a well-characterized pathological hallmark of AD pathogenesis, plays a critical role [12]. Microglia, which act as the primary immune sentinels, initiate the inflammatory response by releasing proinflammatory mediators, which activate astrocytes [13]. Reciprocally, astrocytes regulate microglial activity through the secretion of molecules such as GM-CSF, GDNF or TGF-β, thereby influencing their gene expression and overall response [14]. Additionally, astrocytes provide crucial feedback on the extent of neuronal damage and the efficacy of the ongoing immune response, ultimately contributing to the fine-tuning of microglial activity [15]. This intricate interplay between microglia and astrocytes is essential for maintaining a balanced neuroinflammatory response within the CNS [16, 17] and involves several other cell types and ABC transporters (reviewed in [18]).

One of the mechanisms involved in microglial activation by chronic fibrillary Aβ deposition is the priming and activation of the NLRP3 inflammasome. The NLRP3 inflammasome is a multiprotein complex that plays a critical role in the innate immune response, particularly in the regulation of neuroinflammation [19]. The inflammasome negatively affects the clearance of microglia [10, 20–22] through processes involving phagocytosis, lysosomal damage and cathepsin B release [23]. The sensor protein is the NLRP3 domain, and the adaptor protein is the apoptosis-associated speck-like protein (ASC) containing a CARD domain [24]. Activation and consequent assembly of NLRP3 leads to proximity-induced autocatalysis of pro-caspase-1 to produce mature caspase-1, which then cleaves the inactive proinflammatory cytokines pro-IL1β and pro-IL18 in their secreted forms, IL1β and IL18, respectively [19, 25]. This may contribute to the progression of AD, as has been demonstrated in the murine APPPS1 model [26]. These proinflammatory cytokines bind to specific receptors (TNFR1/2, IL1R1/3, TLR or P2X7R) on astrocyte surfaces, leading to increased production of inflammatory mediators and potentially neurotoxic levels of glutamate [27]. Interestingly, IL1β and TNFα can work together to achieve more robust activation, highlighting the intricate interplay between these molecules in regulating astrocyte function [28]. Understanding this communication pathway is crucial because it holds promise for developing therapies that can modulate astrocyte activity to manage neuroinflammation.

Additionally, it is not yet known whether ABCA7 influences this process and how it might be regulated. Therefore, our group developed the first humanized, Cre-inducible *ABCA7*^*flx*^ mouse model, which was used to generate a conditional microglia-specific ABCA7^ko^ (*Cx3cr1*-hA7^ko^) line. This line was crossbred to the well-established APPPS1-21 β-amyloidosis model to dissect the role of microglial ABCA7 in AD progression and unravel its function in neuroinflammation.

Here, we show that the complete absence of the ABCA7 transporter leads to an increase in soluble and insoluble Aβ levels as well as increased astrogliosis and microgliosis (GFAP^+^ and IBA1^+^), respectively, in a sex-dependent manner, which is positively correlated with the activation of the NLRP3 inflammasome and the release of different proinflammatory cytokines. However, the equivalent levels of Aβ and NLRP3 activation in APPPS1 mice and in a microglia-specific model (APPPS1-*Cx3cr1*-hA7^ko^) both in vivo and in vitro indicate that not only microglia but also astrocytes may be involved in the activation and maintenance of the inflammatory response in our AD mouse model. Taken together, our results highlight an interplay between microglia and astrocytes, possibly mediated by the release of proinflammatory cytokines, that regulates neuroinflammation in AD through ABCA7 function.

## Materials & methods

### Animal models and breeding scheme

The *Abca7*^tm1.1(*ABCA7*)Pahnk^ allele (MGI: 6258226) was bred in homozygosity on the C57BL/6 background (B6J.Cg-*Abca7*^tm1.1(*ABCA7*)Pahnk^). The constitutive knockouts were generated by crossing this line with the Cre-expressing line B6.C-Tg^*CMV−cre*^1Cgn/J (JAX, strain 006054). The resulting heterozygous knockouts were bred back to homozygosity by negatively selecting the Tg^*CMV−cre*^1Cgn allele until extinction. The floxed-allele and the knockout-bearing lines were independently crossed to the APPPS1-21 line (B6.Cg-Tg^Thy1−APPSw, Thy1−PSEN1*L166P^/21JkcrPahnk). In both cases, the *Abca7* allele (either floxed or knocked out, named APPPS1-hA7^flx^ and APPPS1-hA7^ko^, respectively) was further bred back to homozygosity, whereas the APPPS1 transgene (Tg^Thy1−APPSw, Thy1−PSEN1*L166P^/21Jckr) was maintained at hemizygosity (+/Tg). APPPS1-21 (named APPPS1) mice have combined APP (Swedish mutation) and PS1 (L166P mutation) transgenes under the control of the *Thy1* promoter, leading primarily to pathological Aβ production in fronto-cortical neurons and the first cortical Aβ plaques at 45–50 days of age [29]. The constitutive knockouts were generated by crossing individuals with the APPPS1 transgene and the floxed *Abca7*^tm1.1(*ABCA7*)Pahnk^ allele (*hA7*) to the B6.129P2(C)-*Cx3cr1*^tm2.1(creERT2)Jung/^J line (JAX, strain 020940) (named APPPS1-*Cx3cr1*-hA7^ko^). Once more, the floxed *Abca7*^tm1.1(*ABCA7*)Pahnk^ allele was bred back to homozygosity. In experimental animals treated with tamoxifen, the *Cx3cr1*^*creERT2*^ targeted mutation (*Cx3cr1*^tm2.1(*cre/ERT2*)Jung^) was present in heterozygosity (+/tm). As breeders, the sires were always selected to carry the APPPS1 transgene, whereas the *Cx3cr1*^*creERT2*^ mutation may be inherited from either the sires or the dams.

The animals were housed at the Department of Comparative Medicine (section Radium Hospital) at the Oslo University Hospital (Norway) at a temperature of 22 ± 1 °C, relative humidity of 62 ± 5%, 15 air changes per hour, and light cycles of 12 h dark/light (strength: 1 lx - night, 70 lx - day, 400 lx - working illumination) in Eurostandard type III cages (Makrolon^®^) filled with aspen wood (*Populus tremula*, Tapvei^®^, Estonia) as bedding substrate and provided with additional enrichment material (tissue paper, tunnel rods and occasionally gnawing sticks). The animals were grouped into cohorts of up to 8 individuals per cage. Mice were fed *ad libitum* (maintenance expanded pellets from SDS, Estonia) and offered water acidified to pH 3. Health monitoring was performed three times per year according to the FELASA guidelines, and opportunists were included in one of the tests. All experiments were conducted following the guidelines for animal experimentation of the European Union and Norwegian national laws.

### Genotyping of mouse *Abca7* and human *ABCA7*

To genotype the new *ABCA7* locus, we designed primers for the identification of the recombinant, *wild-type* and knockout sequences (Table S1). PCR cycling was performed as follows: 5 min at 95 °C; 35 cycles of 45 s – 95 °C, 60 s – 62 °C, and 90 s – 72 °C; 5 min of elongation of the final products at 72 °C; and cooling at 4 °C until further use. The genotyping PCR showed a clear differentiation between all possible genotypes: homozygous *ABCA7* recombinant (tm/tm), heterozygous *Abca7/ABCA7* recombinant (+/tm), constitutive *Abca7* knockout (−/−, ko), *Abca7* haplodeficient (+/−), and *wild-type* (+/+) animals (Figure S1).

### Tamoxifen treatment

The tamoxifen induction technique involves the use of a microglia-specific Cre recombinase (*Cx3cr1-*CreERT2), which can be activated by tamoxifen through the known Cre/loxP site-specific recombination system and the generation of a specific microglial ABCA7 transporter. Tamoxifen was applied twice within 48 h at the age of 4 weeks. Hereby, an emulsion of 4 mg of tamoxifen (originator AstraZeneca, purchased at Merck KGa, Darmstadt, Germany) in 200 µL of corn oil (Merck KGaA) was injected subcutaneously in four 50 µL depots. The injection was carried out under general anesthesia (sevoflurane 3.5% in an oxygen/nitrogen mixture).

### Tissue collection and processing

Mice were euthanized by a ketamine/xylazine overdose of 400 mg/kg ketamine (VetViva Richter GmbH, Wels, Austria) and 40 mg/kg xylazine (Bayer, Barmen, Germany) at 100 and 200 days of age. After intracardial perfusion with ice-cold PBS, the brains were removed and separated into two hemispheres. One hemisphere was kept in paraformaldehyde (PFA 4% in PBS for immunohistology), and the other hemisphere was snap frozen in liquid nitrogen and later transferred to − 80 °C (for protein and RNA extraction).

### Proteomics analyses using LC-MS/MS

Brain tissue samples from APPPS1 and APPPS1-hA7^ko^ mice (*n* = 14 / 15) mice were used as biological replicates for proteomics analyses. For each replicate equal amounts of brain homogenate (appr. 20 µg of protein) were precipitated on amine beads as previously described [30]. The precipitated proteins on beads were dissolved in 50 mM ammonium bicarbonate, reduced, alkylated and digested with trypsin (1:50 enzyme: protein ratio; Promega, USA) at 37 °C overnight. Digested peptides were acidified and desalted on EVOTIPs using a standard protocol from EVOSEP (EVOSEP Biosystems, Denmark). Liquid chromatography with tandem mass spectrometry (LC-MS/MS) analysis was carried out using an EVOSEP one LC system coupled to a timsTOF pro2™ mass spectrometer, using a CaptiveSpray nanoelectrospray ion source (Bruker Daltonic GmbH, Bremen, Germany). 200 ng of digested peptides were loaded onto a capillary C18 column.15 cm length, 150 μm inner diameter, 1.5 μm particle size, EVOSEP, Odense Denmark).). Peptides were separated at 50 °C using the standard 30 sample/day method from EVOSEP. The timsTOF pro2™ mass spectrometer was operated in data-dependent Parallel Accumulation-Serial Fragmentation (PASEF^®^) mode [31]. Mass spectra for MS and MS/MS scans were recorded between m/z 100 and 1700. Ion mobility resolution was set to 0.85–1.40 V·s/cm over a ramp time of 100 ms. Data-dependent acquisition was performed using four PASEF MS/MS scans per cycle with a near 100% duty cycle. A polygon filter was applied in the m/z and ion mobility space to exclude low m/z, singly charged ions from PASEF precursor selection. An active exclusion time of 0.4 min was applied to precursors that reached 20,000 intensity units. Collisional energy was ramped stepwise as a function of ion mobility. Raw data files from LC-MS/MS analyses were submitted to MaxQuant software (version 2.4.3.0, Max-Planck-Institute of Biochemistry, Martinsried, Germany) for protein identification and quantification [32]. The UniProt mouse database (UniProt Consortium, European Bioinformatics Institute, EMBL-EBI, UK) was used. Trypsin without proline restriction enzyme option was used, with two allowed miscleavage sites. Carbamidomethyl was set as a fixed modification and acetyl (protein N-term), carbamyl (N-term) and oxidation (M) were set as variable modifications. First search peptide tolerance of 20 ppm and main search error 4.5 ppm were used. The allowed FDR was 0.01 (1%) for peptide and protein identification. Label-free quantitation (LFQ) was employed with default settings.

### Isolation of CD11b^+^ microglial cells

Microglia were isolated from 100-day-old APPPS1 and APPPS1-hA7^ko^ mice. Briefly, brains were homogenized in a glass potter with dissection buffer (1x HBSS, 45% glucose, 1 M HEPES). After centrifugation, the pellet was resuspended in 70% Percoll, which was covered with a layer of 30% Percoll. The gradient was centrifuged (45 min, 800 × g, 4 °C), and myelin debris in the interphase between 25% Percoll (Cytiva Freiburg, Germany) and PBS was removed. The collected cells were resuspended in PB buffer (1x PBS, 0.5% BSA) and centrifuged again (10 min, 300 × g, 4 °C). Once the supernatant was discarded, the pellet was resuspended in PB buffer, and CD11b microbeads (Miltenyi Biotec Norden AB, Lund, Sweden) were added. After 15 min of incubation in the dark and under cold conditions, CD11b^+^ microglia were separated using a DynaMag-2 magnetic separator (Invitrogen, Waltham, MA, USA).

### Quantification of Aβ_42_

The frozen hemispheres were thawed on ice in 500 µL of RNAlater^®^ (Merck KGaA, Darmstadt, Germany) for one hour and homogenized for 60 s with four 2.8 mm ceramic beads (OMNI International, Kennesaw, GA, USA) using a SpeedMill PLUS (Analytik Jena GmbH, Jena, Germany). A total of 20 mg of homogenate was mixed with 10 µl of ice-cold Tris-buffered saline [TBS, pH 7.5, containing protease inhibitor (Roche, Mannheim, Germany)] per 1 mg of brain tissue. The samples were homogenized with 2.8 mm ceramic beads (SpeedMill PLUS, 30 s) and centrifuged (16,000 g, 4 °C, 20 min) to separate soluble and aggregated Aβ. The resulting supernatant (TBS fraction containing soluble Aβ) was collected and stored at − 20 °C until further use. The pellet was mixed with 8 µl of cold 5 M guanidine-HCl buffer (pH 8.0) per 1 mg of brain homogenate and homogenized (SpeedMill PLUS, 30 s). The samples were incubated at room temperature for 3 h under constant shaking (1,500 rpm) before centrifugation (16,000 × g, 4 °C, 20 min). The supernatant (the GuHCl fraction containing aggregated Aβ) was collected and stored at − 20 °C until further use. To quantify Aβ_42_ in TBS/GuHCl fractions and CD11b^+^ cells (diluted in GuHCl buffer), we performed electrochemiluminescence immunoassays using the V-PLEX Plus Aβ_42_ Peptide (4G8) Kit (detection limit above 516 fg/ml) and a MESO QuickPlex SQ120 machine according to the manufacturer’s recommendations (Meso Scale Diagnostics LLC, Rockville, MD, USA) [33–36]. The results were normalized to the sample weight. The brain Aβ_42_ concentration was calculated as pg/mg brain.

### Immunohistochemistry and morphological quantification

Formalin-fixed hemispheres were embedded in paraffin and cut into 4-µm-thick coronal sections using a rotation microtome (HM355S, Leica Biosystems GmbH, Nussloch, Germany). Sections at bregma + 0.8 mm and − 1.8 mm were stained for Aβ (anti-human Aβ clone 4G8; 1:4000, BioLegend, USA), microglia (anti-IBA1, 1:1,000, FUJIFILM Wako Chemicals Europe GmbH, Germany, 019-19741) and astrocytes (anti-GFAP, 1:500, Agilent, USA, Z033401-2) using a BOND-III^®^ automated immunostaining system (Leica Biosystems GmbH, Nussloch, Germany) with a hematoxylin counterstain (provided with the staining kit, Bond Polymer Refine Detection, DS9800) [37, 38]. After staining, tissue sections were digitized at 230 nm resolution per pixel using a Pannoramic MIDI II slide scanner (3DHISTECH Ltd., Budapest, Hungary) [39]. Quantitative analysis was performed automatically using deep learning algorithms generated with DeePathology™ STUDIO (DeePathology Ltd., Ra’anana, Israel) using algorithms previously established by the laboratory [40, 41]. The cell/plaques density (number per ROI), relative area coverage (% cell area per total ROI), and cell size were determined for each animal.

### Real-time RT‒qPCR analysis

Total RNA was extracted from primary cell culture pellets or brain tissue homogenates using an RNeasy Mini Kit and QIAzol as a lysis reagent (Qiagen, Hilden, Germany). The total amount of RNA extracted was quantified with a NanoDrop 1000 (Thermo Fisher, Waltham, MA, USA) by absorbance at 260 nm, and its purity was calculated as the ratio between the absorbance values at 260/280 nm and 230/260 nm. cDNA was synthesized from 1 µg of total RNA using the commercial RNeasy Mini QuantiTect Reverse Transcription Kit (Qiagen, Hilden, Germany). Quantitative real-time PCR assays were performed using TaqMan Gene Expression Assays (Applied Biosystems, Foster City, CA, USA) to quantify the mRNA levels of *Nlrp3* (Mm00840904_m1), *Pycard* (Mm00445747_g1), *Casp1* (Mm00438023_m1), *Tlr4* (Mm00445273_m1), *Cd36* (Mm00432403_m1), *Nfkb1* (Mm00476361_m1), *P2rx7* (Mm01199500_m1), *Ctsb* (Mm01310506_m1), and *Gapdh* (Mm99999915_g1) as endogenous controls for normalization. Quantitative PCR was performed using the StepOne Plus Real Time PCR System (Applied Biosystems, Foster City, CA, USA), and the threshold cycle (Ct) was calculated by the instrument’s software (v2.4, Sequence Detection, Applied Biosystems, Foster City, CA, USA). The expression levels were calculated using the 2^−ΔΔCt^ method.

### Western blot analyses

For protein extraction, 30 mg of brain homogenate tissue was lysed in ice-cold RIPA buffer (50 mM Tris-HCl, pH 8; 150 mM sodium chloride; 1 mM EDTA; 1% Triton X-100; 0.5% sodium deoxycholate; 0.1% sodium dodecyl sulfate) supplemented with complete protease inhibitor cocktail (Roche, Mannheim, Germany). The final protein concentration in the supernatants was determined using a Pierce BCA assay kit (Thermo Fisher, Waltham, MA, USA), and a total of 25 µg of protein was subjected to 4 − 15% Mini-PROTEAN polyacrylamide gel electrophoresis (Bio-Rad Laboratories, Hercules, CA, USA) and transferred onto a polyvinylidene difluoride (PVDF) membrane (Bio-Rad Laboratories) using a mini Trans-Blot Electrophoretic Transfer Cell (Bio-Rad Laboratories). The membranes were then blocked for 1 h at room temperature with Tris-buffered saline containing 5% nonfat dry milk and 0.1% Tween-20 and incubated with anti-caspase1 (1:1,000; clone EPR16883, Abcam, Cambridge, UK, ab179515) and anti-beta-tubulin (1:2,500; clone AA2, MerckMillipore, Burglinton, MA, USA, T5076) antibodies overnight at 4 °C. The membranes were finally incubated with an enhanced chemiluminescence (ECL) horseradish peroxidase-conjugated secondary antibody (1:5,000, Bethyl Laboratories, Montgomery, TX, USA, A90-216P) for 1 h at room temperature. Images were obtained by using the Octoplus QPLEX imaging system after the membranes were incubated with Clarity Max ECL substrate reagent (Bio-Rad Laboratories).

### Quantification of cytokines

Brain homogenates were extracted using lysis buffer (phosphate-buffered saline + 1% Triton X-100 + phosphatase inhibitor + protease inhibitor) at 2 µL/mg brain homogenate. The samples were homogenized for 30 s (SpeedMill PLUS, Analytic Jena GmbH, Jena, Germany) and centrifuged at 16,000 × g for 40 min at 4 °C. Supernatants were diluted 4-fold (for brain tissue samples) or 2-fold (for cell culture medium) using a working solution (Meso Scale Diagnostics LLC, Rockville, MD, USA), and immunoassays were performed using the V-PLEX Proinflammatory Panel 1 (mouse) Kit (detection limit above 40 fg/ml) and a MESO QuickPlex SQ120 machine following the manufacturer’s instructions.

### Primary glial cell cultures

Pure astrocyte or microglial cell cultures were obtained from APPPS1 and APPPS1-hA7^ko^ pups (P0-P5), respectively, using the protocol previously published by Güler et al. [42] with some modifications (Figure S4A). In brief, brains were collected in 1× HBSS buffer (Gibco, Grand Island, NY, USA) and homogenized with DNase I (Merck KGaA, Darmstadt, Germany) and 0.05% trypsin-EDTA (Thermo Fisher Scientific - Gibco, Grand Island, NY, USA) after meningeal removal. After centrifugation (10 min, 150 × g), the pellet was resuspended in DMEM (for astrocytes) or DMEM/F12 (for microglia) (Gibco, Grand Island, NY, USA) supplemented with 10% FBS (Cytiva, Freiburg, Germany) and 2% penicillin/streptomycin (Gibco, Grand Island, NY, USA) and transferred to a T-25 flask that was incubated in a humidified atmosphere of 5% CO_2_ at 37 °C. When the cultures reached confluence (7 − 10 days), the OLs were removed by flask tapping, and the remaining cells were removed from the flask using 0.05% trypsin-EDTA. After centrifugation (10 min, 150 × g), the pellet containing a mixture of microglia and astrocytes was resuspended in complete DMEM or DMEM/F12, and the cells were separated following consecutive incubation steps on sterile bacterial-grade plates. When the derived APPPS1-hA7^ko^ microglial cultures reached confluence (≈ 2 × 10^6^ cells), they were incubated in DMEM/F12 supplemented with 1% FBS. Three hours later, the cells were treated with 100 ng/ml LPS (from *E. coli* 055:B5, Merck KGaA - Sigma‒Aldrich, San Louis, MS, USA) for 24 h. Then, the media was removed to be added to the APPPS1 astrocyte cultures (≈ 2 × 10^6^ cells) to induce an inflammatory reaction via the conditioned media. Twenty hours after the addition of conditioned media stimulated with LPS, astrocyte and microglia pellets and medium were collected for qPCR and cytokine ELISA, respectively.

### Statistical analysis

All the statistical analyses were performed using GraphPad Prism software (v9, Dotmatics, Boston, MA, USA). We verified that the data had a normal Gaussian distribution by using the Shapiro‒Wilk normality test. If all groups were normally distributed (*p* > 0.05), we performed one-way ANOVA with Tukey’s correction for multiple comparisons. Student’s t test was also performed to determine the significant differences between the two experimental groups. The data are presented as the means ± standard deviations (SD). Differences were considered statistically significant when *p* < 0.05. The number of animals used in each experiment is reported in the figure legends. LFQ proteomic data were analyzed using Perseus (v2.0.11, https://cox-labs.github.io/coxdocs/perseus_instructions.html) [43] and VolcaNoseR (https://github.com/JoachimGoedhart/VolcaNoseR) [44], which plot the fold change (log_2_ transformation) *versus* the *p* value (− log_10_ transformation) for all the quantified proteins. This generates a so-called volcano plot, which shows a measure of effect size *versus* a measure of significance. The data points with the largest effect size and a statistical significance threshold > 1.3 (corresponding to *p* < 0.05) were considered hits. These protein hits are annotated in the plot with an abbreviated name (mouse nomenclature).

## Results

### Generation of a mouse line with humanized, floxed ABCA7 expression (*ABCA7*^flx/flx^)

**Fig. 1:**
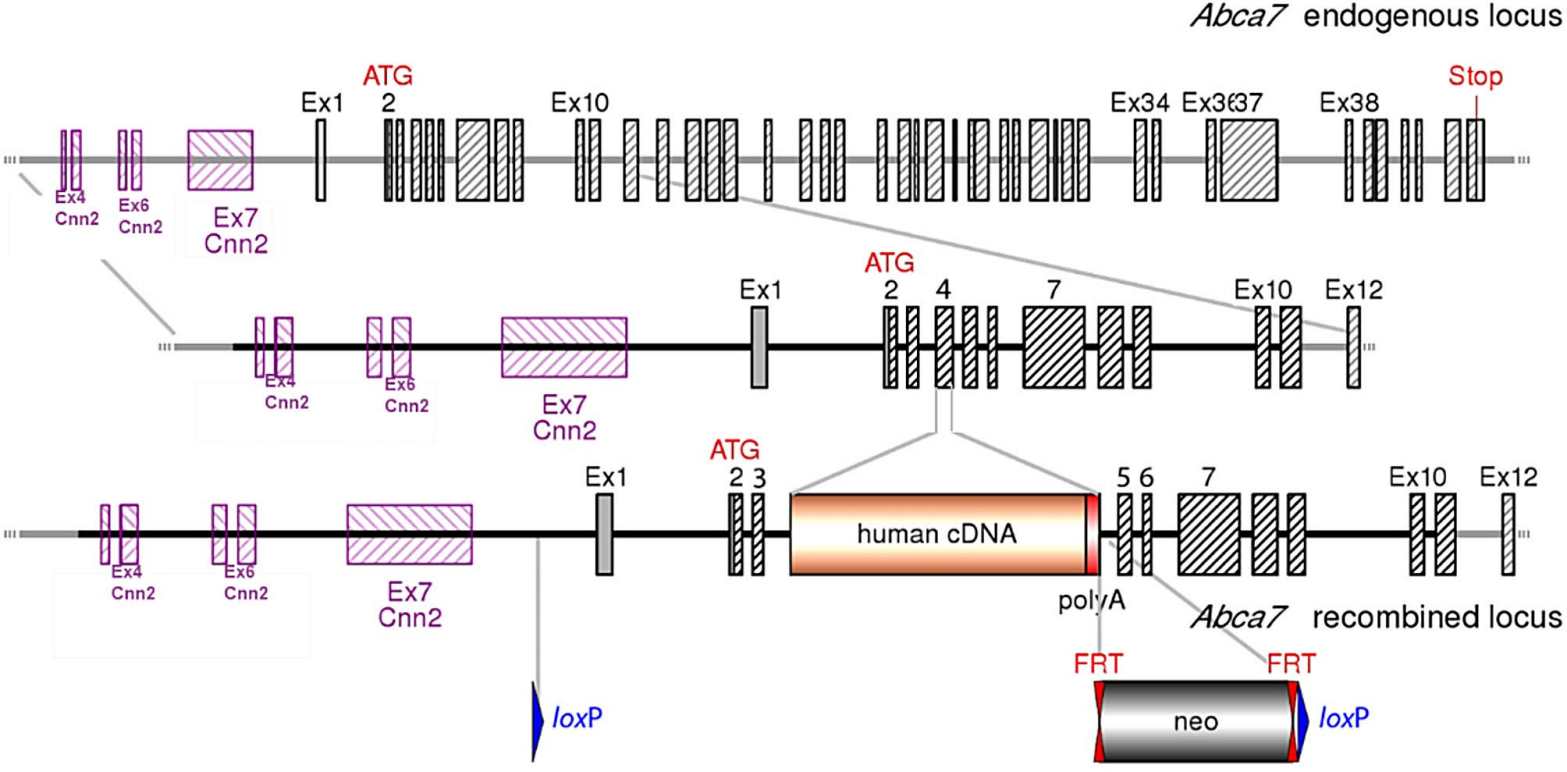
Generation of a humanized ABCA7 mouse strain (*ABCA7*^flx/flx^). In-frame insertion of human type I *ABCA7* cDNA starting from exon 2 at the 5’ end of mouse *Abca7* exon 4. An exogenous polyadenylation sequence (human growth hormone polyA) was inserted downstream of the human *ABCA7* stop codon. This produces a mouse/human chimeric ABCA7 protein with an N-terminal portion encoded by mouse *Abca7* exons 2 and 3, while the remaining C-terminal sequence is encoded by the inserted human *ABCA7* cDNA. This approach avoids putative deregulation of the neighboring *Cnn2* gene and results in disruption of the mouse gene. In addition, *loxP* sites are inserted upstream of *Abca7* exon 1 and downstream of the humanized *ABCA7* cDNA cassette, allowing conditional deletion of the human gene

The design and generation of the new humanized ABCA7 mouse line were performed in close collaboration with genOway SA (Lyon, France). Humanization of the murine *Abca7* gene locus was accomplished by inserting human type I *ABCA7* cDNA starting from human exon 2 into the 5’ end of mouse *Abca7* exon 4 (Table S2). Thus, we avoided deregulation of neighboring genes while disrupting the *Abca7* murine gene locus. Additionally, two loxP sites were inserted upstream of *Abca7* exon 1 and downstream of the *ABCA7* cDNA. The insertion of these sites enables the targeted deletion of *ABCA7* cDNA using a Cre recombinase, generating conditional or constitutive knockout models (Fig. 1). The vector was purified and inserted into C57BL/6 N embryonic stem (ES) cells by electroporation. The allele was registered as *Abca7*^tm1.1(*ABCA7*)Pahnk^ in the Mouse Genome Informatics database (MGI:6258226).

**Fig. 2:**
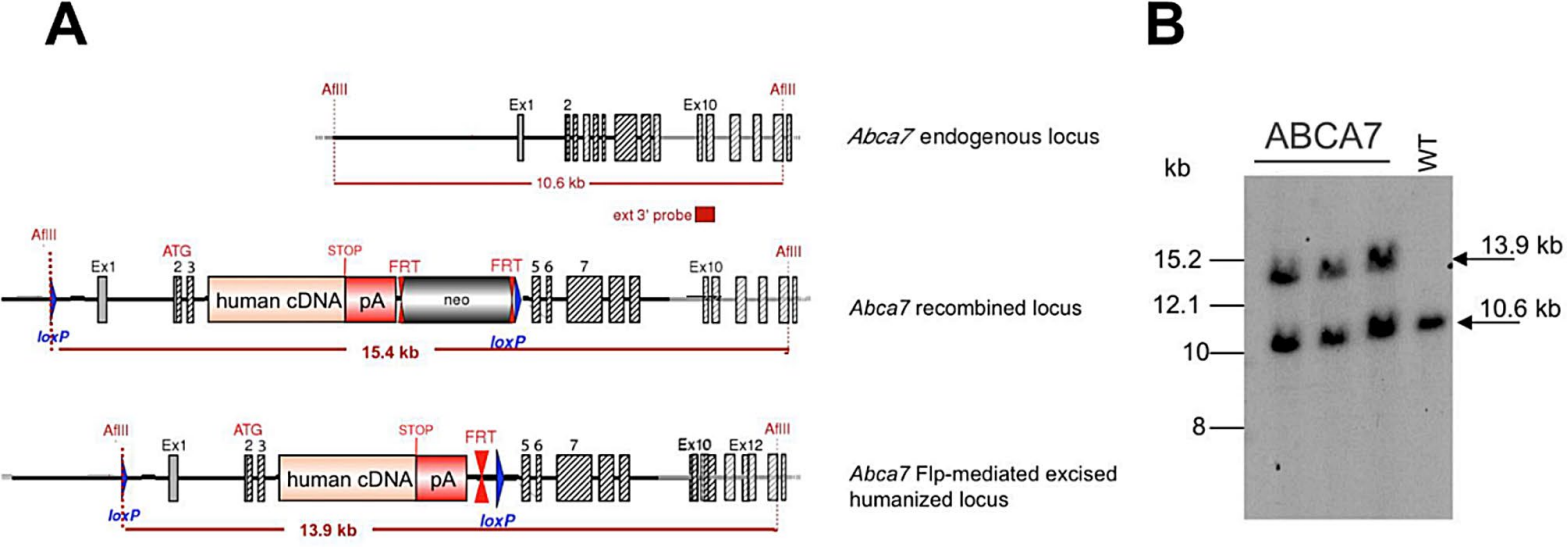
Southern blot analysis of heterozygous, neo-excised, humanized ABCA7 mice. Schematic representation of the expected alleles for Southern blot and genotyping analyses: *Abca7* wild-type (10.6 kb), recombinant (15.4 kb) and Flp-mediated, neo-excised (13.9 kb) humanized *ABCA7* and Cre-excised *ABCA7/Abca7* knockout (**A**). The genomic DNA of the tested heterozygous, neo-excised, humanized *ABCA7* animals (*n* = 3) showed the expected 13.9 kb band, and wild-type C57BL/6J DNA showed only the 10.6 kb band (**B**)

ES cell clones were selected and assessed for correct homologous recombination and insertion of the *ABCA7* cDNA. To validate the insertion of *ABCA7* cDNA in the targeted *Abca7* locus at exon 4 and assess the deletion of the ABCA7 insert in Cre-recombined knockout mice (quality control test), Southern blot analyses for homolog and induced recombination were performed with tissue samples from Abca7 *wild-type* controls, *ABCA7*^flx/flx^, and ABCA7^ko^ mice (Fig. 2).

### Effects of ABCA7 knockout on amyloid-β burden and neuroinflammation

**Fig. 3:**
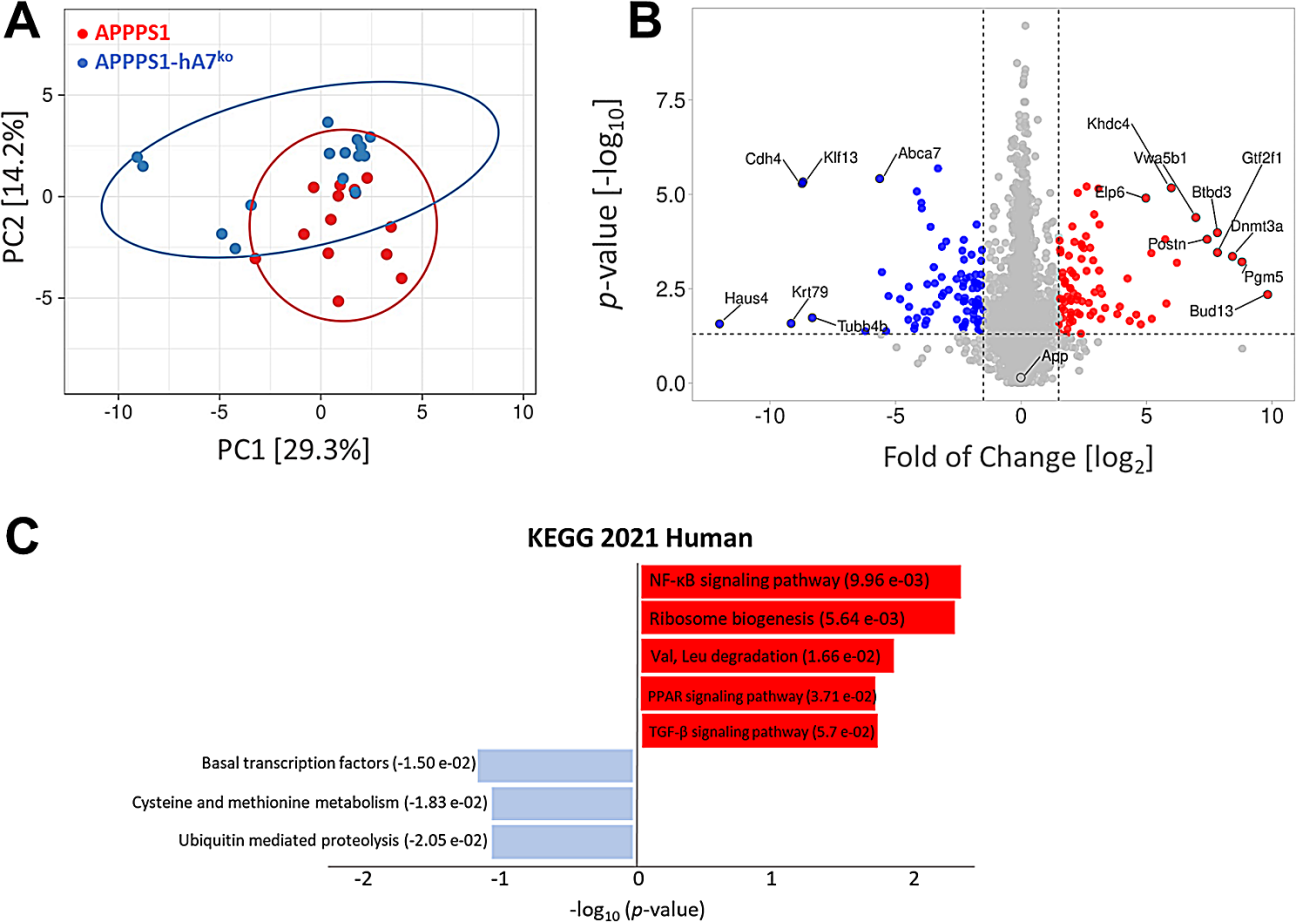
Proteome analysis of brain tissue samples from APPPS1-hA7^ko^and APPPS1 mice. PCA plot showing the percentage of variability between samples due to the genotype effect (**A**). LFQ-based volcano plot showing the changes in protein expression in brain tissue between APPPS1-hA7^ko^ (*n* = 15) and APPPS1 (*n* = 14) mice. The horizontal dotted line represents a significance level of *p* = 0.05 (–log_10_ = 1.3), and the vertical dotted lines represent a fold change ± 1.5 (log_2_)(**B**). KEGG pathway analysis (*KEGG human 2021*) was conducted to detect pathways differentially regulated among protein hits detected in LFQ-volcano plot data. Red bars represent the most upregulated pathways, while blue bars represent the most downregulated ones. (**C**)

To investigate the impact of the deletion of the humanized ABCA7 transporter in our AD model, we analyzed the brain proteome of chimeric APPPS1-hA7^ko^ (*n* = 8 males, 7 females) and APPPS1 mice carrying the wild-type mouse *Abca7* gene (*n* = 7 males, 7 females) from 50 to 200 days of age by LC‒MS/MS (DDA). Principal component analysis (PCA), which defines the variation within experimental groups, revealed that 29.3% of the variability between APPPS1-hA7^ko^ and APPPS1 was due to the lack of the ABCA7 transporter, separating the mice into two distinct clusters (Fig. 3A). Based on the volcano plot data, we found a total of 166 differentially expressed proteins in APPPS1-hA7^ko^ mice compared to controls, with log_2_-fold change values between ± 1.5 and significance values (− log_10_) greater than 1.3 (corresponding to *p* = 0.05). Among all hits, 84 proteins were upregulated and 82 proteins were downregulated in the absence of the humanized ABCA7 transporter (Fig. 3B).

Proteomic hits were classified according to pathway involvement using the *KEGG 2021 Human database* embedded in the *Enrichr* bioinformatics resource [45–47]. We found that hABCA7^ko^ induced the upregulation of several proteins involved in NFκB signaling, one of the canonical pathways involved in the control of inflammation, among other processes. Dysregulation of ubiquitin-related proteolysis and the TGFβ signaling pathway was also found, which might be closely related to the toxic aggregation of Aβ and the consequent apoptotic process (Fig. 3C).

Next, we performed unbiased functional enrichment analysis (Gene Ontology, GO) of the differentially expressed proteins found in the volcano plot using the *Enrichr* database. In this case, the absence of the ABCA7 transporter in the APPPS1 mouse model was associated with changes in biological processes related to inflammation control and regulation. Positive regulation of cellular senescence (GO: 2000774; *p* value = 0.00029) was the most enriched biological process. The inflammatory response is one of the hallmarks of cellular senescence, and it serves to both propagate the senescence process and facilitate its clearance [48, 49]. A fairly recent publication identified the expression of senescence-related genes specifically in TREM2^+^ microglia in the 5xFAD β-amyloidosis model, highlighting the link between these two degenerative processes [50]. Other biological processes enriched and directly related to the modulation of the immune response in the CNS were the IL1-mediated signaling pathway (GO: 0070498; *p* value = 0.00105) and the positive regulation of T-cell cytokine production (GO: 0002726; *p* value = 0.00128). The enrichment of these biological processes could be related to increased cytokine release, which also affects other cellular functions. One example is the negative regulation of mTOR signaling (GO: 0032007; *p* value = 0.00100), which is involved in the control of other key cellular mechanisms that are altered in AD, such as autophagy or reactive oxygen species (ROS) generation (Table 1).

**Table 1:** Enriched biological processes (Gene Ontology terms) in APPPS1-hA7^ko^ mice

GO term	Biological process	Adjusted *p* value
2000774	positive regulation of cellular senescence	0.00029
1901184	regulation of ERBB signaling pathway	0.00049
0032801	receptor catabolic process	0.00066
0032007	negative regulation of mTOR signaling	0.00100
0016180	snRNA processing	0.00105
0070498	interleukin-1 (IL1)-mediated signaling pathway	0.00105
0002726	positive regulation of T-cell cytokine production	0.00128
0034260	negative regulation of GTPase activity	0.00128

Based on the proteome findings, we investigated whether ABCA7 is a key player in neuroinflammation control in AD. To this end, we measured Aβ_42_ levels in CD11b^+^ microglia isolated from the brains of 100-day-old APPPS1 expressing the wild-type ABCA7 protein and chimeric APPPS1-hA7^ko^ mice. While the absence of the transporter in female mice leads to a significant decrease in Aβ_42_ levels, in APPPS1-hA7^ko^ males, the levels of Aβ_42_ were increased compared to those in APPPS1 control mice (Figure S2). This data show how ABCA7 excerts sex-specific roles in microglial Aβ clearance. Whereas in males its absence seems to be protective by increasing the uptake of the toxic peptide, in females there is a loss of the transporter’s protective role when it is functional.

**Fig. 4:**
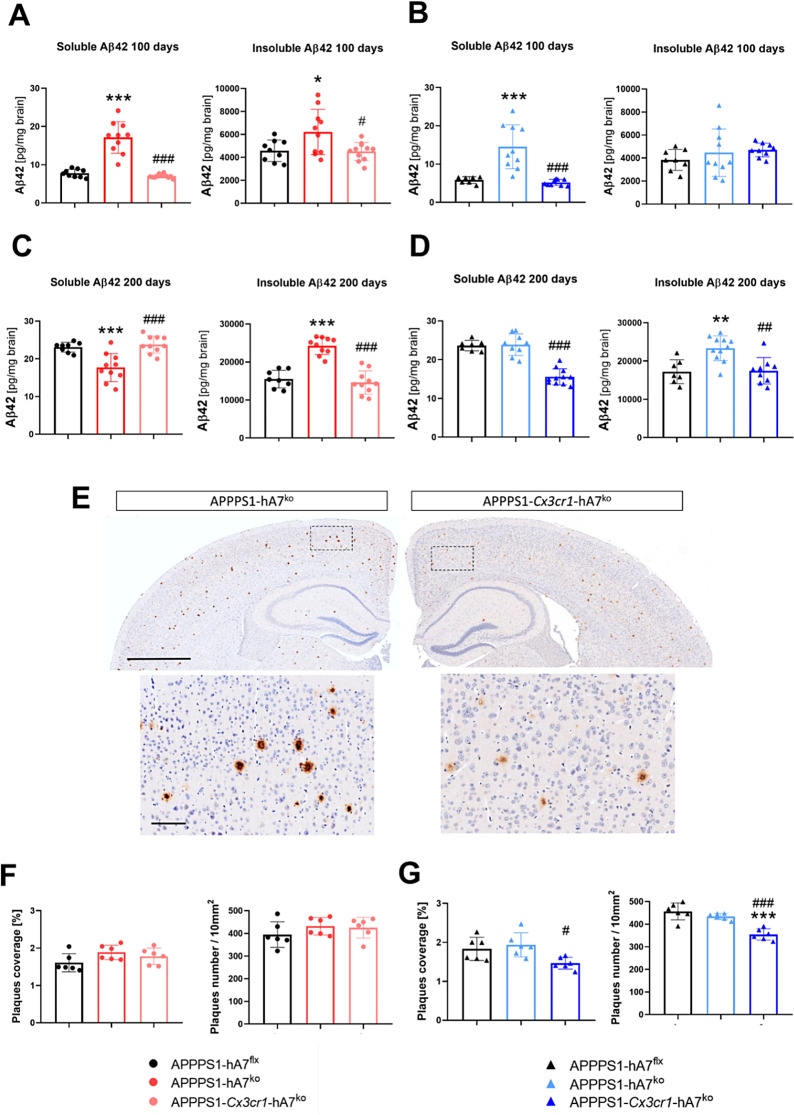
Effect of ABCA7^ko^* versus* conditional *Cx3cr1-ABCA7*^ko^ on Aβ load and deposition in APPPS1 mice during disease progression. Quantitation of soluble and insoluble Aβ_42_ levels in females and males at 100 (**A**, **B**) or 200 days of age (**C**, **D**). Representative images of Aβ immunostaining on brain sections from 100-day-old male mice (**E**). Plaque coverage and number of amyloid plaques were determined in both females (**F**) and males (**G**). The data are presented as the means ± SDs (*n* = 9–10/experimental group); significance was determined using one-way ANOVA followed by Tukey’s *post hoc* test (*p ≤ 0.05, **p ≤ 0.01, ***p ≤ 0.001 vs. APPPS1-hA7^flx^; # *p* ≤ 0.05, ## *p* ≤ 0.01, ### *p* ≤ 0.001 vs. APPPS1-hA7^ko^)

To further investigate the link between microglia and the ABCA7 transporter, we developed a conditional knockout model of the ABCA7 transporter in Cx3cr1^+^ microglia, inducible by tamoxifen treatment, crossbred with the APPPS1 model. From these breedings, we finally got APPPS1-hA7^flx^ as the control group, the chimeric APPPS1-hA7^ko^ mice and the conditional APPPS1-*Cx3cr1*-hA7^ko^ mice. First, we checked the status of β-amyloidosis by measuring Aβ_42_ levels. Human Aβ_42_ is preferentially generated over Aβ_40_ in our AD mice model [29]. By immunoassay, we observed that soluble Aβ_42_ increased in APPPS1-hA7^ko^ mice at 100 days of age compared to that in age-matched APPPS1-hA7^flx^ controls, but in the conditional *Cx3cr1*-hA7^ko^ group peptide levels returned to control APPPS1-hA7^flx^ levels in both males and females (Fig. 4A-B). This indicate that selective loss of the ABCA7 transporter in microglia does not affect total soluble Aβ_42_ levels, even when microglial Aβ clearance it is affected, as has been described before (Figure S2). Sex differences are also evident in insoluble peptide levels. In females, aggregated Aβ_42_ increased in APPPS1-hA7^ko^ mice and returned to control levels in APPPS1-*Cx3cr1*-hA7^ko^ mice, whereas in males, there were no significant differences between the experimental groups (Fig. 4A-B). Analysing highly aggregated Aβ in plaques, the number and coverage is significantly different in male mice (Fig. 4G). The complete absence of the ABCA7 transporter didn’t change Aβ plaque coverage and number in comparision to APPPS1-hA7^flx^ control mice, whereas conditional knockout on Cx3CR1 microglia cells showed a significant reduction in both parameters demonstrating the functional role that microglial ABCA7 may exert for Aβ load and the formation of Aβ plaques (Fig. 4F-G). It is noteworthy that the decrease on insoluble Aβ_42_ quantify on conditional *Cx3cr1*-hA7^ko^ mice by immunoassay also correlates to the number and to the density of the plaques observed by IHC (Fig. 4E). At 200 days of age, when β-amyloidosis is widespread throughout the brain (the total amount of insoluble Aβ_42_ detected is approximately 4-fold greater than that detected at 100 days), immunoassay measurements showed that complete ABCA7^ko^ protects against soluble Aβ_42_ burden but not conditional knockout in microglia in females (Fig. 4C). Concerning insoluble Aβ, which at this stage is more toxic and physically associated with the microglia that surround the plaques, APPPS1-*Cx3cr1*-hA7^ko^ was again able to control the levels of these peptides in both male and female mice (Fig. 4C-D).

**Fig. 5:**
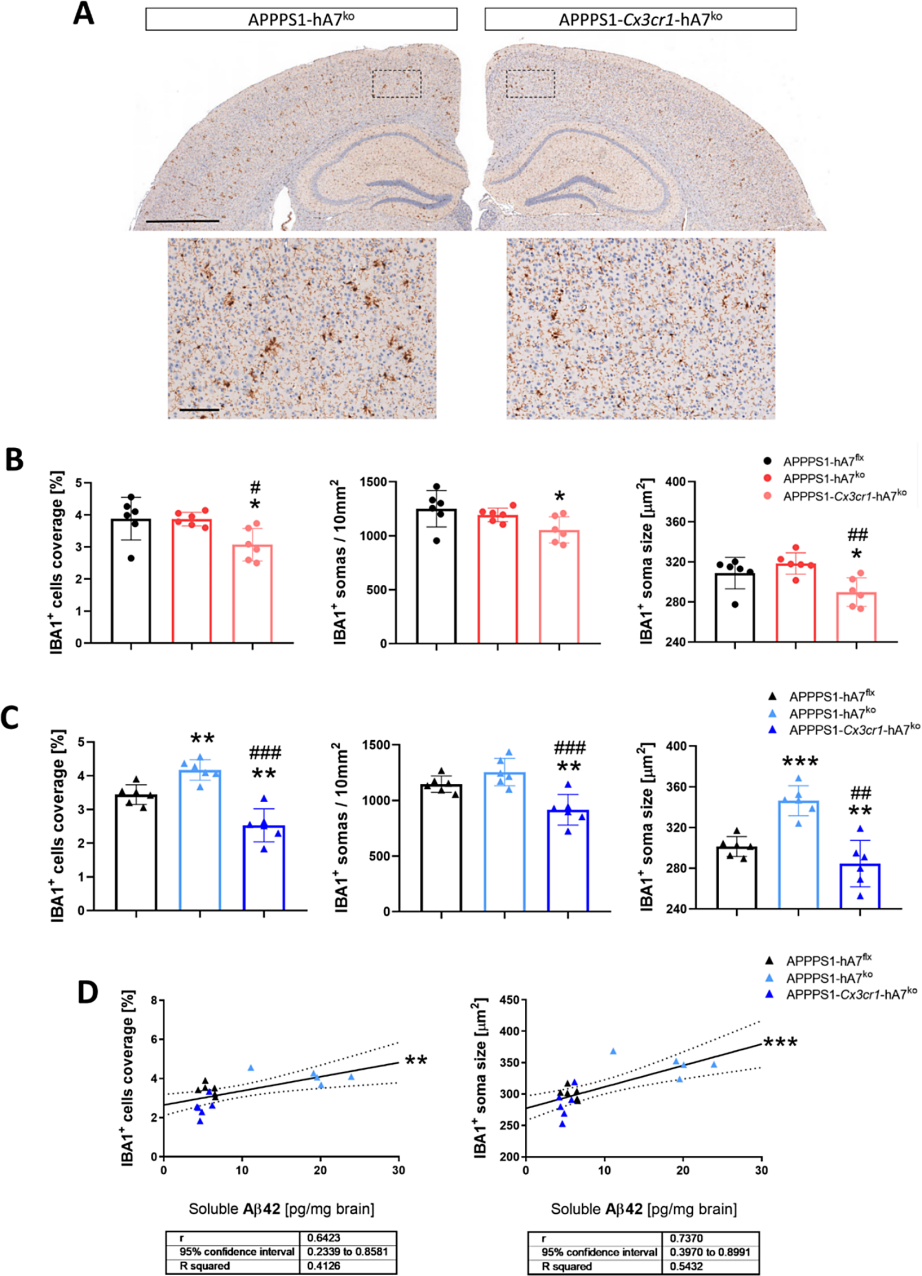
Morphological changes in microglia induced by ABCA7^ko^*or* conditional *Cx3cr1*-ABCA7^ko^ in 100-day-old APPPS1 mice. Representative images of IBA1 immunostaining in brain sections from 100-day-old male mice (**A**). Microglial activation was determined by the density of IBA1^+^ cells, the number of IBA1^+^ cells, and the soma size of IBA1^+^ cells in females (**B**) and males (**C**). Correlations between soluble Aβ_42_ levels (TBS fraction) and Iba1 cell coverage or IBA1^+^ cell soma size in males (**D**). The data are presented as the means ± SDs (*n* = 6 per experimental group); significance was determined using one-way ANOVA followed by Tukey’s *post hoc* test (*p ≤ 0.05, **p ≤ 0.01 vs. APPPS1-hA7^flx^; # *p* ≤ 0.05, ## *p* ≤ 0.01, ### *p* ≤ 0.001 vs. APPPS1-hA7^ko^). Scale bars indicate 1,000 μm (overviews) or 100 μm (details)

**Fig. 6:**
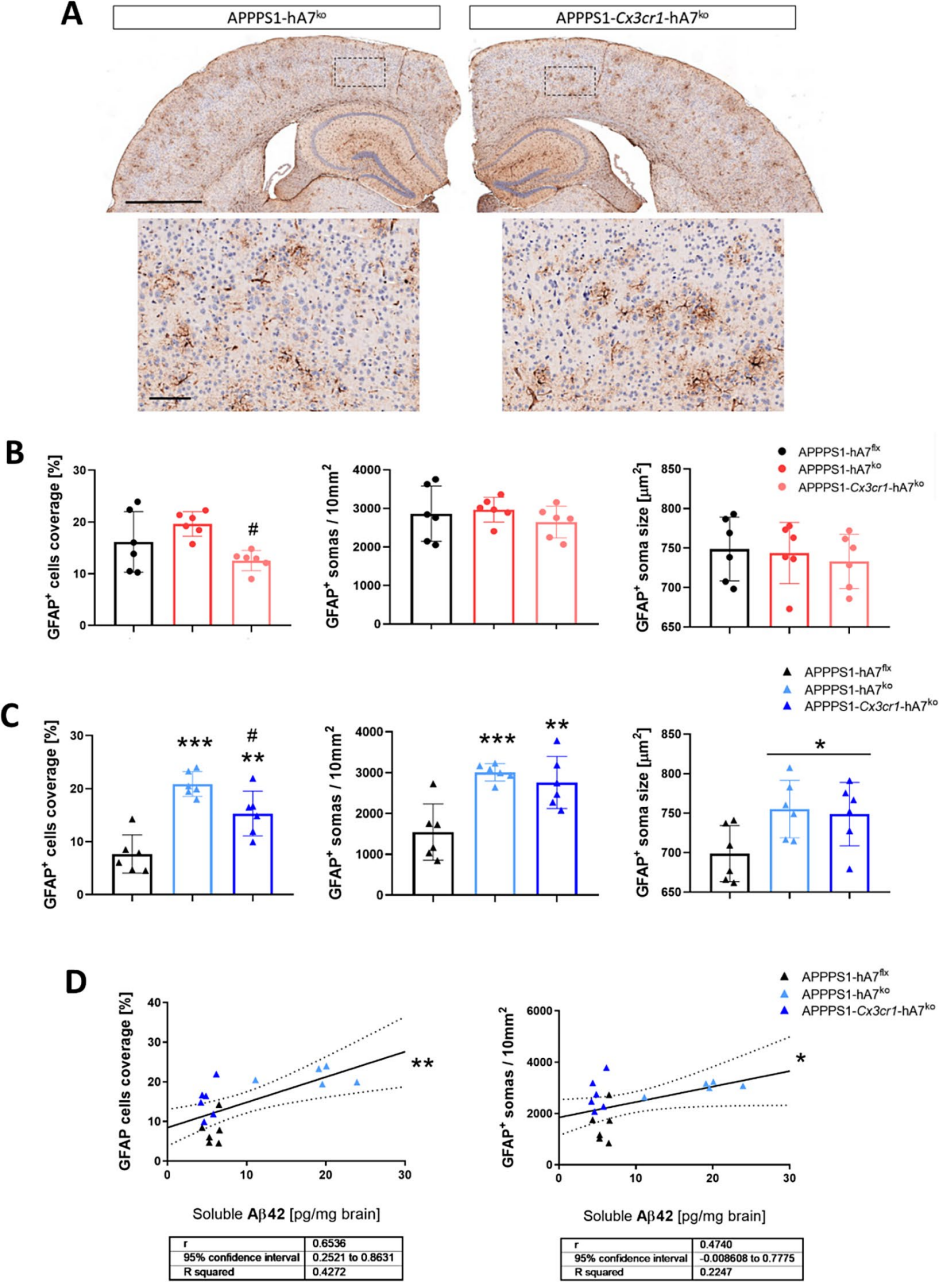
Morphological changes in astrocytes induced by ABCA7^ko^* or* conditional *Cx3cr1-ABCA7*^ko^ in 100-day-old APPPS1 mice. Representative images of GFAP immunostaining of brain sections from 100-day-old male mice (**A**). Astrocyte activation was determined using the density of GFAP^+^ cells, the number of GFAP^+^ cells and the soma size of GFAP^+^ cells in females (**B**) and males (**C**). Correlations between soluble Aβ_42_ levels (TBS fraction) and GFAP cell coverage or GFAP^+^ cells in males (**D**). The data are presented as the means ± SDs (*n* = 6/experimental group); significance was determined using one-way ANOVA followed by Tukey’s *post hoc* test (**p ≤ 0.01; ***p ≤ 0,001 vs. APPPS1-hA7^flx^; # *p* ≤ 0.05 vs. APPPS1-hA7^ko^). Scale bars indicate 1000 μm (overviews) or 100 μm (details)

The status of microglia and astrocytes in the brain was examined by IHC at 100 days of age, at which point the changes in β-amyloidosis status were pronounced and physiologically translated to the onset and development of the disease in AD patients. Regarding microglial IBA1^+^ cells, we observed that IBA1 coverage and cell size in APPPS1-hA7^ko^ female mice were the same as those in APPPS1-hA7^flx^ control mice, whereas ABCA7 transporter knockout in CX3CR1^+^ cells reduced microglial cell coverage and their possible activation state (Fig. 5B). In males, the complete absence of the ABCA7 transporter leads to a large increase in IBA1^+^ cell coverage and soma size, which could be related to the development of a neuroinflammatory phenotype. This effect was restored to control levels when the knockout was restricted to microglial CX3CR1^+^ cells in the conditional model in both males and females (Fig. 5A-C). We also checked whether there was a link between microgliosis in male mice and β-amyloidosis. A clear positive correlation existed between soluble Aβ_42_ (not an aggregated peptide), IBA1 coverage and the size of microglia (Fig. 5D), revealing how the microglial phenotype could change in response to increased amyloid production. Higher levels of Aβ_42_ in APPPS1-hA7^ko^ male mice are associated with the presence of brain microgliosis, whereas lower levels of Aβ_42_ are associated with reduced microglial coverage and soma size in the APPPS1-hA7^flx^ and APPPS1-*Cx3cr1*-hA7^ko^ groups. This positive correlation was also detected in males (but not in females) because higher levels of soluble Aβ_42_ peptide are associated with pronounced astrogliosis, which was more evident in the APPPS1-hA7^ko^ group (Fig. 6D).

GFAP changes are restricted to males; in females, we did not observe any significant differences. This could be related to the general increase in all parameters evaluated in the female APPPS1-hA7^flx^ control group compared to the male group (Fig. 6B). Both APPPS1-hA7^ko^ and APPPS1-*Cx3cr1*-hA7^ko^ males showed a greater percentage of astrocytic brain coverage and a greater total number of GFAP^+^ astrocytes than did the APPPS1-hA7^flx^ controls (Fig. 6C). These parameters are the main hallmarks of astrogliosis in vivo and could be caused by morphological changes such as hypertrophy of the main cellular processes or loss of the filamentous shape of the cell, among others. The IHC results suggest that neuroinflammation observed in the APPPS1 model could be driven, at least in part, by the ABCA7 transporter, which is expressed not only in astrocytes but also in microglia. ABCA7-related communication could be essential for the modulation of this pathogenic mechanism.

### Evaluation of NLRP3 inflammasome status and cytokine profiles in vivo and in vitro

**Fig. 7:**
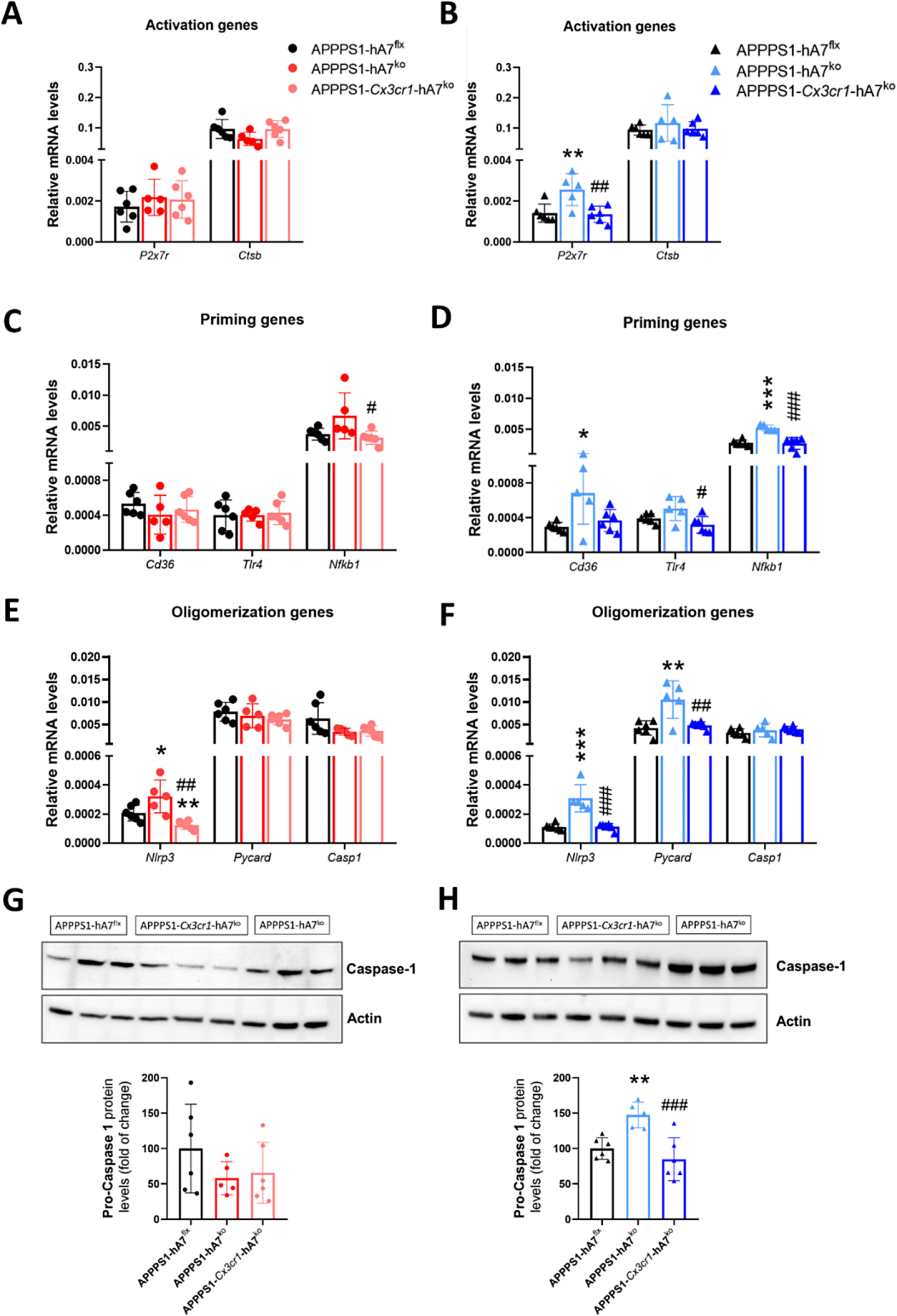
Changes in the NLRP3 inflammasome induced by ABCA7^ko^* versus* conditional *Cx3cr1-ABCA7*^ko^ in 100-day-old APPPS1 mice. mRNA expression levels quantified by qPCR of genes involved in the activation (**A**, **B**), priming (**C**, **D**), and oligomerization (**E**, **F**) of the NLRP3 inflammasome system in both males and females. Western blot analysis of caspase-1 protein levels in females (**G**) and males (**H**). The data are presented as the means ± SDs (*n* = 5 − 6/experimental group); significance was determined using one-way ANOVA followed by Tukey’s *post hoc* test (*p ≤ 0.05, **p ≤ 0.01, ***p ≤ 0.001 vs. APPPS1-hA7^flx^; # *p* ≤ 0.05, ## *p* ≤ 0.01, ### *p* ≤ 0.001 vs. APPPS1-hA7^ko^)

One of the most studied mechanisms of inflammatory signaling in glial cells is the NLR-based inflammasome, whose main cellular response is the release of proinflammatory cytokines and pyroptosis [51]. We used qRT‒PCR to determine the mRNA expression levels of the different players involved in the sequential steps required for the triggering of this complex, including activation, priming, and oligomerization genes, in brain tissue samples from 100-day-old mice. As we have previously observed in the IHC results, most mRNA changes occur in male mice. In females, we did not detect differences between the experimental groups (Fig. 7A, C, E). In contrast, there was a clear increase in the expression of the *P2rx7*, *Cd36* and *Nfkb1* genes in APPPS1-hA7^ko^ male mice compared to APPPS1-hA7^flx^ controls. However, conditional knockout of the ABCA7 transporter in CX3CR1^+^ microglia restored the mRNA expression of all these markers to the APPPS1-hA7^flx^ control group levels (Fig. 7B-D) which indicates that selective loss of the ABCA7 transporter in microglia does not affect NLRP3 activation and priming, while complete loss of ABCA7 does.

According to the literature, Aβ can trigger the activation of CD36 and P2X7 receptors [19]. We verified that in male mice, we found a significant positive correlation between soluble Aβ_42_ levels quantified by immunoassay and the mRNA expression levels of these genes (Figure S4A-B). This is an indirect way of showing how the β-amyloidosis deposition process might influence the neuroinflammatory process in the APPPS1 model. Regarding the genes directly involved in the assembly of the inflammasome complex, we detected increased mRNA expression of *Nlrp3* and *Pycard*, but not of *Casp1*, in APPPS1-hA7^ko^ male mice, which was reversed to control levels in the absence of the transporter on microglia (Fig. 7F). One of the signals that initiates the assembly of NLRP3 into the ASC protein is the activation of the NFκB pathway. There was a positive correlation between the mRNA expression levels of *Nfkb1* and *Nlrp3* in both males and females, with the highest levels in APPPS1-*Cx3cr1*-h7^ko^ mice (Figure S4C-D).

Even if the expression of the *Casp1* gene was not altered, we wanted to determine whether the levels of the functional protein responsible for converting pro-IL1β to the active form of this cytokine were dysregulated. In females, we did not observe any change in the qPCR data (Fig. 7G), but in males, we noticed the same trend as in the qPCR results: an increase in caspase-1 protein in the APPPS1-hA7^ko^ group, which returned to control levels in the APPPS1-*Cx3cr1*-hA7^ko^ mice. (Fig. 7H) We also detected NLRP3 inflammasome activation at 200 days of age, but in this case, we did not observe any differences (Figure S3). These results are consistent with our previous findings and suggest that ABCA7 functionality may be essential for regulating the inflammatory state in the brain via modulation of the NLRP3 inflammasome complex at early stages of the disease.

**Fig. 8:**
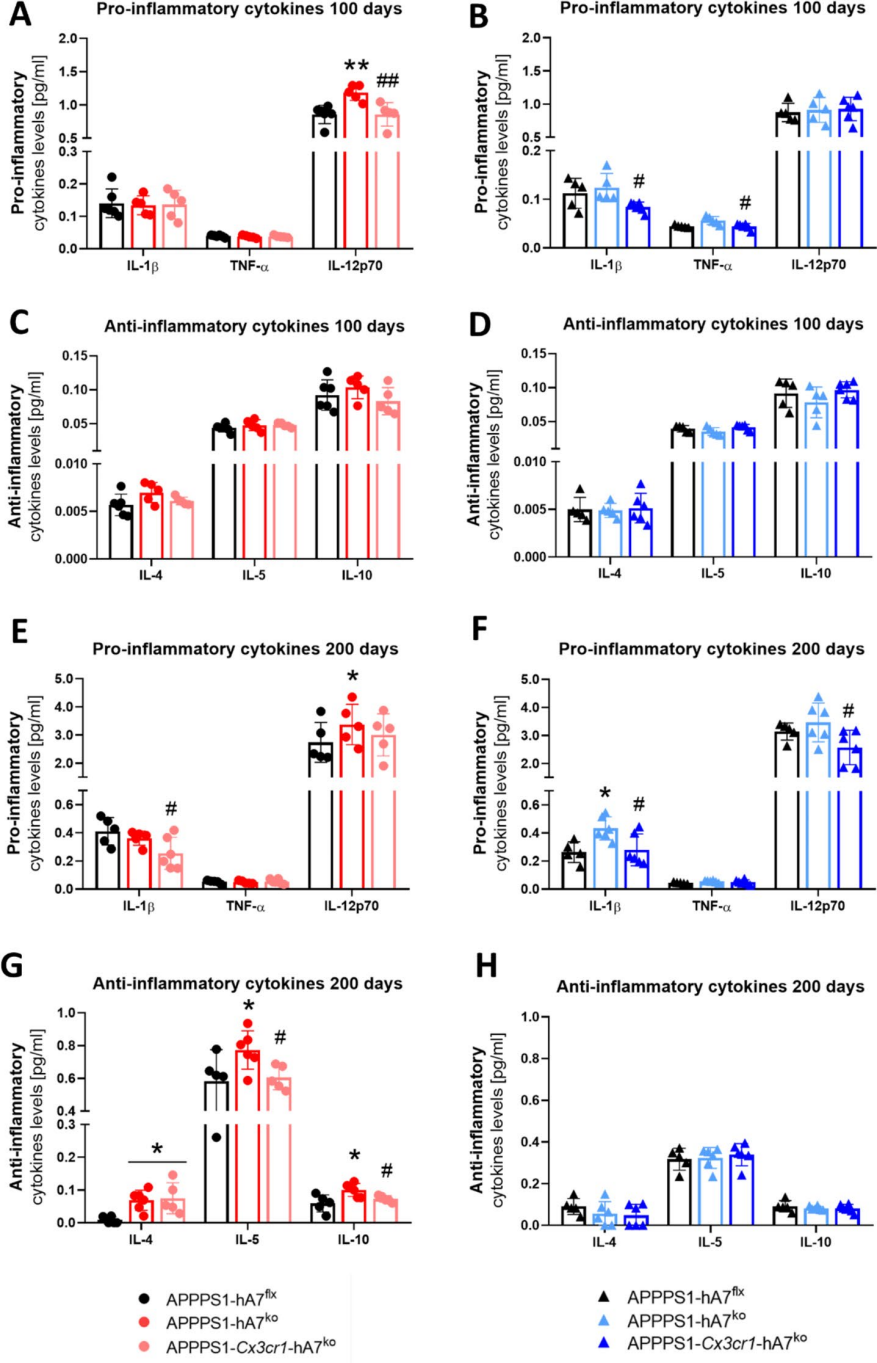
Changes in cytokine release induced by ABCA7^ko^* versus* conditional *Cx3cr1-ABCA7*^ko^ in APPPS1 mice during disease progression. Levels of the proinflammatory cytokines IL1β, TNFα and IL12p70 quantified by ELISA in females and males at 100 (**A**, **B**) and 200 days of age (**E**, **F**). Levels of the anti-inflammatory cytokines IL-4, IL-5 and IL-10 quantified by ELISA in females and males at 100 (**C**, **D**) and 200 days of age (**G**, **H**). The data are presented as the means ± SDs (*n* = 5–6 per experimental group); significance was determined using one-way ANOVA followed by Tukey’s *post hoc* test (*p ≤ 0.05, **p ≤ 0.01 vs. APPPS1-hA7^flx^; #*p* ≤ 0.05, ## *p* ≤ 0.01 vs. APPPS1-hA7^ko^)

To gain a complete overview of the inflammasome complex and its activation, we also measured the protein levels of selected cytokines in brain tissue samples using immunoassays. At 100 days of age, we did not observe any marked changes in females, except for an increase in IL12p70 (Fig. 8A). In males, the results are consistent with the qRT‒PCR data. We detected a decrease in the levels of IL1β and TNFα in the APPPS1-*Cx3cr1*-hA7^ko^ mice, which was consistent with the decrease in caspase-1 protein levels and the expression of NLRP3-related genes (Fig. 8B). No changes in the levels of anti-inflammatory cytokines were detected at this age (Fig. 8C-D). At 200 days, when the system collapsed due to the burden and deposition of Aβ, changes in cytokine levels became more evident. In females, there was an increase in the anti-inflammatory cytokines IL-4, IL-5 and IL-10 in the APPPS1-hA7^ko^ group, which was restored to control levels by the absence of the transporter only in microglia (Fig. 8G>). This increase in anti-inflammatory cytokines may be related to the greater ability of female mice to counteract neuroinflammation than male mice, in whom ABCA7 deficiency appears to have a detrimental effect. Concerning proinflammatory cytokines, we quantified a decrease in the levels of IL1β and TNFα in both male and female APPPS1-*Cx3cr1*-hA7^ko^ mice (Fig. 8E-F).

We were also interested in how microglia lacking the functional ABCA7 transporter were able to induce changes in astrocytes exposed to the expression of the APP transgene under the control of the Thy1 promoter. For this purpose, we performed an in vitro conditioned culture assay on postnatal pups (P0-P5) in which we treated APPPS1 astrocytes with conditioned media from APPPS1-hA7^ko^ microglia to assess the communication between these two cell types. First, we verified the purity of both primary cell cultures by qPCR for *Gfap* (enriched in astrocytes) and *Cx3cr1* (enriched in microglia) markers, which were within the expected levels (Figure S5B).

**Fig. 9:**
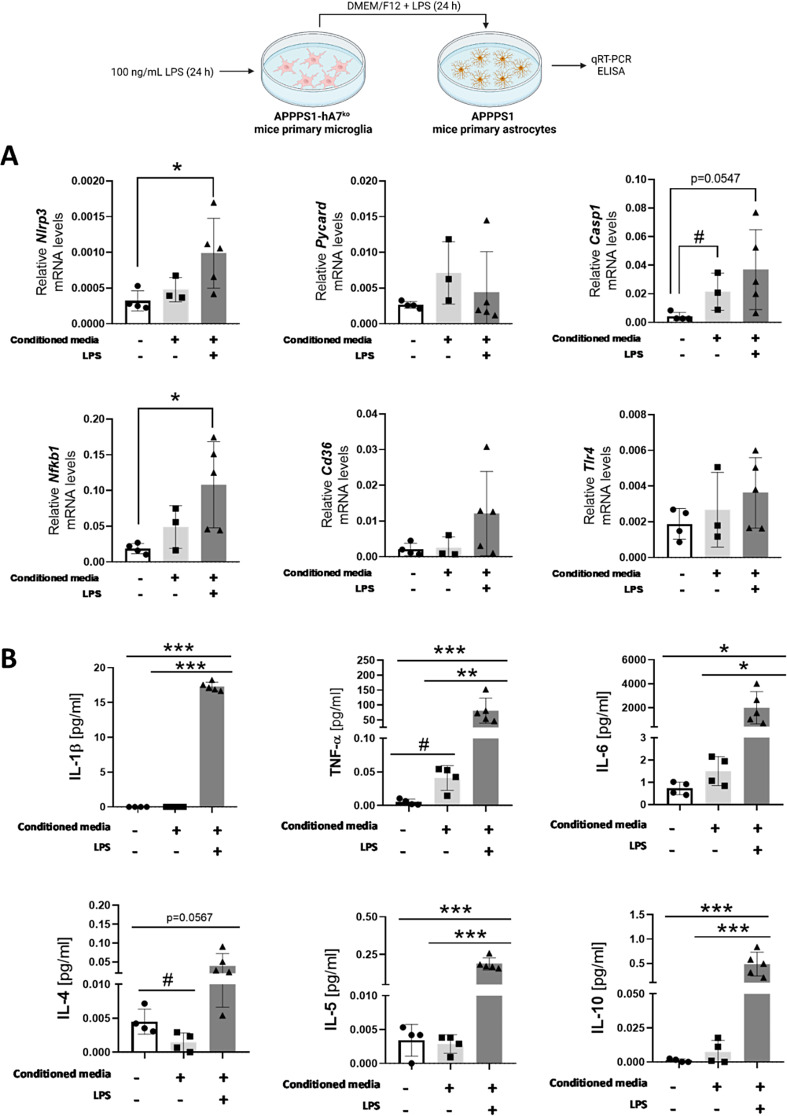
APPPS1-hA7^ko^ microglia induce NLRP3 inflammasome expression in APPPS1 astrocytes in vitro. mRNA expression levels quantified by qPCR of genes involved in the priming and oligomerization of the NLRP3 inflammasome in APPPS1 + astrocytes treated with conditioned medium from APPPS1-hA7^ko^ mice for 24 h (**A**). Levels of selected proinflammatory/anti-inflammatory cytokines quantified by ELISA in APPPS1 astrocytes treated with conditioned medium from APPPS1-hA7^ko^ mice for 24 h (**B**). The data are presented as the means ± SDs (*n* = 3–5 independent cultures per experimental condition); significance was determined using one-way ANOVA followed by Tukey’s *post hoc* test (*p ≤ 0.05, **p ≤ 0.01, ***p ≤ 0.001 vs. conditioned medium (+), LPS (+) group; #*p* ≤ 0.05 vs. conditioned medium (+), LPS (−) group)

We established two experimental groups of APPPS1*-*hA7^ko^ mice to mimic in vitro the same inflammatory conditions observed in vivo: (a) untreated microglia or (b) microglia stimulated with 100 ng/mL LPS for 24 h. LPS stimulation led to increased expression of NLRP3 inflammasome assembly genes (Figure S5C) as well as a significant increase in proinflammatory cytokine release (IL1β, TNFα, IL-6 and IL12p70) in APPPS1-hA7^ko^ microglia (Figure S5D). Conditioned media from both groups of microglia was used to stimulate APPPS1 astrocytes for another 24 h. qRT‒PCR measurements showed that APPPS1-treated astrocytes incubated in the presence of LPS(+)-conditioned media (named APPPS1 (+ LPS)) expressed higher levels of *Nlrp3*, *Casp1* and *Nfkb1* than control DMEM-treated astrocytes or astrocytes incubated with conditioned media from nontreated microglia (named APPPS1 (− LPS)). In this case, there was a trend that did not reach statistical significance. (Fig. 9A). These results were consistent with the cytokine measurements. APPPS1 astrocytes (+ LPS) release increased levels of the proinflammatory cytokines IL1β, TNFα, IL12p70 and IL-6 as well as some anti-inflammatory cytokines, such as IL-4, IL-5 and IL10, into the media. The incubation of astrocytes with untreated microglial cell media significantly increased the levels of some of these cytokines (Fig. 9B), indicating that LPS is required in these early developmental conditions to induce the inflammatory conditions observed in adult mice. The in vitro data mimic the results obtained in vivo and support the existence of an interplay between microglia and astrocytes in the absence of the ABCA7 transporter, which may be involved in modulating the neuroinflammatory response.

## Discussion

Compared with traditional transgenic mice, humanized mice are becoming an increasingly valuable tool in AD research due to their ability to better mimic human conditions [52, 53]. This advantage stems from two key features. First, humanized mice can be engineered to express genes associated with human AD, such as the Aβ protein. This allows the effects of these human proteins to be studied directly in living organisms, bridging the gap between genetic mutations and their effects on the brain [54]. Second, humanized mice can be bred to represent the genetic diversity observed in human populations [53], which is particularly relevant to LOAD, the most common form of the disease. Indeed, in 2011, the *ABCA7* transporter locus was identified as the second most important genetic risk factor after *APOE4* for sporadic LOAD [3], underlying the importance of this protein in the onset and development of the disease. Our laboratory has previous experience in the development of humanized models based on ABC transporters with knockout capabilities, such as ABCB1 [55] or ABCC1 [56]. We decided to further develop the first humanized, floxed *ABCA7* knock-in mouse crossbred with the amyloidogenic model APPPS1-21 to investigate how changes in this ABC transporter might affect Aβ metabolism and contribute to neuroinflammation in AD.

Interestingly, the ABCA7 transporter has dual functions against the toxic effects of Aβ. It acts as a cellular guardian, shuttling Aβ out of brain cells and preventing its accumulation. However, the influence of ABCA7 may also extend upstream, potentially regulating the processing pathway that generates Aβ [57]. This dual functionality positions ABCA7 as a promising target for therapeutic strategies aimed at combating AD by enhancing ABCA7 efflux activity or modulating its ability to regulate APP processing [5]. Previous studies have shown that in vitro knockout of *ABCA7* induces a decrease in Aβ efflux across the blood‒brain barrier, reducing the clearance of this peptide and facilitating its accumulation in the CNS [58]. In the J20 AD mouse model, knockout of *ABCA7* doubled insoluble Aβ levels and thioflavin S-positive plaques in the brain [59]. Our results are in line with these previous findings and confirm the neuroprotective function of ABCA7 in APP processing. Here, we show that complete absence of the ABCA7 transporter in the β-amyloidosis mouse model APPPS1 induces an increase in fibrillar and insoluble Aβ_42_ levels in both males and females at 100 and 200 days of age. The molecular results also correlate with the extent and number of insoluble Aβ plaques found in the APPPS1-hA7^ko^ group at 100 days. This modulation of APP processing is related not only to ABCA7 but also to other ABC transporters, such as ABCC1. ABCC1 deficiency in the APPPS1 mouse model promotes Aβ deposition and accumulation in the walls of brain microvessels. In contrast, activation of ABCC1 by the agonist thiethylperazine significantly reduced the Aβ load in APPPS1 mice expressing ABCC1 but not in those lacking the ABCC1 transporter [60].

**Fig. 10:**
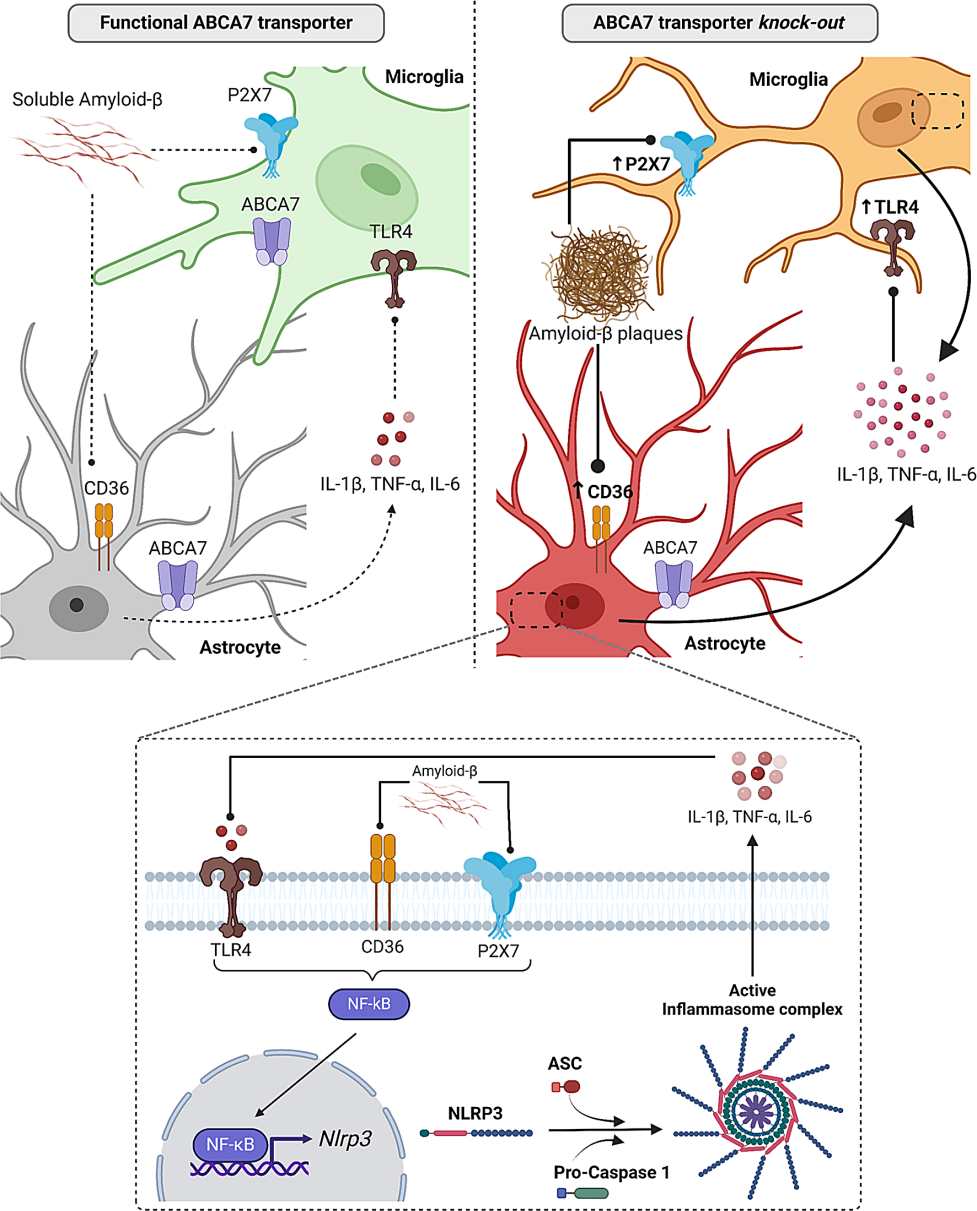
Proposed role of the ABCA7 transporter as modulator of neuroinflammation *via* NLRP3 inflammasome in APPPS1 mice. Representation of NLRP3 inflammasome system activation in astrocytes and microglia with (left panel) and without (right panel) functional ABCA7 transporters. Lines with arrows represent cytokine and mediator release to the extracellular space, and lines with dots indicate receptor activation. Bold lines represent increased cytokine release/receptor activation, and dotted lines reflect basal or decreased cytokine release/receptor activation

In addition to its role in modulating APP processing, the ABCA7 transporter is increasingly recognized as a potential modulator of neuroinflammation. Previous studies have shown that impaired ABCA7 function in microglia results in reduced cholesterol clearance, which may contribute to the accumulation of Aβ peptides observed in AD [10, 61]. In addition, ABCA7 expression appears to influence the activation state of microglia, as lower ABCA7 levels have been observed to correlate with a more proinflammatory phenotype by impairing CD14 expression [10]. These findings suggest a multifaceted role for ABCA7 in microglial function, potentially influencing both Aβ deposition and inflammatory responses in the brain. To investigate this link, we generated a tamoxifen-induced, conditional knockout of *ABCA7* in microglia (*Cx3cr1-*hA7^ko^) (the main results and proposed mechanism are summarized in Fig. 10). We observed that the absence of this transporter in microglia returned to control APPPS1-hA7^flx^ levels of soluble and insoluble Aβ_42_ peptide in both males and females, demonstrating that microglia are not the only cells directly involved in the toxic processing and deposition of Aβ_42_ in the APPPS1 AD model. Analysis of IBA1^+^ cells showed that complete knockout of the ABCA7 transporter in the APPPS1 model increased microglial brain coverage, cell number and soma size (only in males). The microgliosis observed in the APPPS1-hA7^ko^ group may not only be related to chronic Aβ peptide exposure [62] but also to alterations in cholesterol efflux. The absence of ABCA7 reduces high-density lipoprotein (HDL), which has been proposed to inhibit inflammatory signaling through TLR4 suppression in microglia and monocytes [63], supporting the protective role of this transporter in the CNS. Moreover, conditional ABCA7 knockout (*Cx3cr1-*hA7^ko^) not only reverted all these morphological parameters to those of the APPPS1-hA7^flx^ control group but also further reduced them. As we observed on isolated APPPS1-hA7^ko^ microglia, the reuptake and clearance of Aβ was increased, which could be related to a protective microglial phenotype consistent with the IBA1^+^ IHC data, as well as to the decreased levels of some pro-inflammatory cytokines (IL-1β and TNF-α). Moreover, a recent publication describes the generation of a microglial *Abca7*^V1613M^ mutant crossed with a 5xFAD AD model, confirming the relevance of the ABCA7 transporter in microglial cells. This in vivo model showed a reduced response to inflammation, lipid metabolism, Aβ pathology and neuronal damage, demonstrating that this microglial ABCA7 variant may confer a gain of function and provide a protective effect against AD-related pathology [64]. Thus, astrocytes are suspected to be possible promoters of the neuroinflammatory process observed in the APPPS1 model. Astrocytes respond to inflammatory signals and can promote inflammation by secreting cytokines and chemokines, thereby controlling immune cell activation and migration to sites of damage [65]. Therefore, it is not surprising that the communication between astrocytes and CNS-resident or CNS-infiltrating cells plays a central role in tissue pathology even when the mechanisms involved are not completely understood [66]. Our IHC data show that removal of ABCA7 in the APPPS1 mouse model leads to an increase in GFAP^+^ astrocyte brain coverage, cell number and soma size in males, all of which are hallmarks of astrogliosis [67], but not in females.The levels of these markers did not return to control levels in microglia with conditional knockout of *ABCA7* (*Cx3cr1-*hA7^ko^), highlighting the role of ABCA7 in astrocytes. The IHC data confirm that ABCA7 is regulated in a cell-dependent manner, as previously suggested by other authors [68, 69], but the underlying mechanism about why GFAP is increased in conditional ABCA7 knockout mice remain unclear. For example, the reactive astrocyte phenotype A1 characterize by high levels of GFAP promotes inflammation via the NF-kB pathway [70]. One of the genes involved in this signaling cascade, *Nfkb1*, is increased in APPPS1-hA7^ko^ but not in conditional ABCA7 knockout (*Cx3cr1-*hA7^ko^) mice. Further morphological studies as well as single cell RNA-seq and metablome analyses may help to decipher the precise nature of astrocytes in these circumstances. We can state from our available data that the interplay between astrocytes and microglia may play a pivotal role in neuroinflammation, as observed in our AD mouse model.

One molecular mechanism linking excess Aβ in the brain to the activation of neuroinflammation in AD is the NLRP3 inflammasome. In 2013, Heneka et al. described the activation of the NLRP3 inflammasome in the APPPS1 model through an increase in NLRP3 and caspase-1 protein levels in microglia. It increases Aβ deposition by reducing their phagocytic capacity and thus induces a self-perpetuating positive feedback loop. A deficiency of both proteins largely delays memory decline, improves the clearance of Aβ plaques and reduces the levels of IL1β [26, 71]. The absence of ABCA7 has the opposite effect: an increase in Aβ_42_ levels in CD11b^+^ microglia is a result of an increase in their phagocytic capacity, suggesting that ABCA7 is required to modulate NLRP3 inflammasome activation. The underlying mechanisms are not fully understood, but some recent publications have suggested that Aβ may bind to the microglial TLR4/CD36 complex or the TREM2 receptor to trigger downstream signaling cascades, leading to the translocation of the NFκB transcription factor to the nucleus and the consequent release of proinflammatory cytokines such as IL1β, TNFα or IL-6 [20, 21, 72]. Another protein suggested to be involved in the activation and priming of NLRP3 is the P2X7 receptor, which is activated by changes in intracellular K^+^ and Ca^2+^ levels, ROS production and mitochondrial depolarization and induces NFκB translocation to the nucleus to promote the transcription of the *Nlrp3* gene [73]. In our APPPS1-hA7^ko^ model, the quantified increase in soluble and insoluble Aβ_42_ could be directly related to the increased mRNA expression of the main genes involved in the transcription and assembly of the inflammasome complex, such as *Nfkb1*, *Nlrp3* or *Pycard* (genes encoding the ASC protein). Other genes involved in the priming and activation of this protein complex, such as *Tlr4*,* Cd36* and *P2rx7*, were also found to be upregulated in the absence of the ABCA7 transporter. These data show that ABCA7 is involved in the control of NLRP3 inflammasome induction and inflammation in the brain of male mice but not in that of female mice. Conditional knockout of *ABCA7* in Cx3cr1^+^ microglia reverts the expression of all of these markers to control levels. This finding demonstrated that not only microglia are involved in the activation of the NLRP3 inflammasome system.

Crosstalk between astrocytes and microglia during inflammation is mainly mediated by the release of cytokines and chemokines into the extracellular matrix that bind to specific receptors capable of perpetuating inflammatory signals through NFκB activation [27, 74]. A lack of the ABCA7 transporter in microglia in the APPPS1 model induces changes in the *Nfkb1 and Nlrp3* genes and in the caspase-1 protein, which is consistent with the changes in the levels of the cytokines IL1β and TNFα in the brain. To further investigate the communication loop between the two glial cell types, we performed an in vitro assay to evaluate how cytokines released by APPPS1-hA7^ko^ microglia could induce activation of the NLRP3 inflammasome in APPPS1 astrocytes. Previous studies have shown that exposure of astrocytes to IL1β and TNFα, either directly or from conditioned microglial medium previously stimulated with LPS, activates NFκB. The transcription factor then binds to promoter regions and induces a positive autoregulatory feedback loop not only for IL1β and TNFα but also for other cytokines, such as GM-CSF, IL-6 or IL-8 [28, 75, 76]. In our study, LPS stimulation of APPPS1-hA7^ko^ microglia led to upregulation of the NLRP3 inflammasome and the release of proinflammatory cytokines. Increased levels of IL1β, TNFα and IL-6 were found in APPPS1 astrocyte media after 24 h of exposure to microglia-conditioned media, as well as increased mRNA expression of genes involved in their production, such as *Nfkb1*,* Nlrp3* and *Casp1*. This mechanism has also recently been suggested in depression-like mice and multiple sclerosis models [74, 77], and communication between both cell types mediated by the ABCA7 transporter may be responsible for neuroinflammatory responses *via* NLPR3 inflammasome activation, as observed in vivo.

It is very important to note that sex differences are more than evident in all the results presented in this study. Therefore, the inclusion of both males and females in AD translational research is crucial (2–3:1 risk in women *versus* men) [78] but is especially important regarding ABCA7-related mechanisms. As we have mentioned several times before, ABCA7 is directly involved in cholesterol efflux and transport, and cholesterol is the main substrate for the synthesis of steroid hormones such as testosterone or estrogens [18, 79, 80]. Several studies have demonstrated that hormones can also interfere with neuroinflammatory responses induced by NFκB. The anti-inflammatory activity of estrogens is affected via the ER-α receptor, which has been shown to exert a transcriptional inhibitory effect on NFκB, resulting in decreased cytokine and chemokine production [81, 82]. Our results revealed that the ABCA7 transporter plays a key neuroprotective role in controlling not only the amyloidogenic process but also inflammation in males but not in females. The impact of the absence of the ABCA7 transporter on steroid hormone synthesis, as well as its side effects, could be the cause of these differences, but more research is needed to strengthen this theory in the future. It is clear that by including both males and females in ABCA7 research, we can gain a more detailed understanding of how this gene contributes to AD risk and progression and open the way to developing more effective and personalized therapies.

## Conclusion

Taken together, the results of the present study demonstrate that the ABCA7 transporter plays a key role in modulating both β-amyloidosis and inflammation induced by NLRP3 inflammasome in the APPPS1 mouse model of Alzheimer’s disease. The activation of this protein complex requires the close association of astrocytes and microglia and the release of cytokines, which are the main cellular messengers in the interplay between these two cell types, to mediate the inflammatory response.

## Electronic supplementary material

Below is the link to the electronic supplementary material.

Supplementary Material 1: Table S1. Primer sequences for genotyping of the *Abca7* / *ABCA7* locus. Table S2. Comparison of DNA and protein sequences of murine, human and the in-frame inserted construct. Start codon for translation in the mouse gene is located in exon 2. Fig. S1. Agarose gel showing different PCR fragment sizes for each genotype. Fig. S2. Isolation of CD11b^+^ microglial cells from 100-day-old APPPS1 and APPPS1-hA7^ko^ mice. Fig. S3. NLRP3 inflammasome changes induced by *ABCA7* total knockout versus conditional *Cx3cr1*-knockout in 200-day-old APPPS1 mice. Fig. S4. Correlations between soluble Aβ42 levels (TBS fraction) and mRNA expression of NLRP3 inflammasome activators *P2rx7* (A) and *Cd36* (B). Fig. S5. LPS stimulation induces NLRP3 inflammasome expression and pro/anti-inflammatorycytokines release in APPPS1-hA7^ko^ microglia

## Data Availability

The mass spectrometry proteomics data have been deposited to the ProteomeXchange Consortium via the PRIDE partner repository (http://www.ebi.ac.uk/pride/archive/), with the dataset identifier PXD053250 and the project name “ABCA7 transporter functional role on Alzheimer’s disease onset and progression”. Data from published diagrams, figures, and analysis files are available at https://osf.io/vwq58/ (10.17605/OSF.IO/VWQ58; PahnkeLab-open-access-AD).

## Abbreviations

ABCA7: ATP-binding cassette (ABC) transporter A7
Aβ: Amyloid-β
AD: Alzheimer’s disease
APP: Amyloid precursor protein
ASC: Apoptosis-associated speck-like protein
CNS: Central nervous system
Cx3cr1: Cx3C motif chemokine receptor 1
FAD: Familial Alzheimer’s disease
GWAS: Genome-wide association study
IHC: Immunohistochemistry
ko: Knockout
LC‒MS/MS: Liquid chromatography with tandem mass spectrometry
LFQ: Label-free quantitation
LOAD: Late-onset Alzheimer’s disease
LPS: Lipopolysaccharide
NLRP3: NLR family pyrin domain containing 3
PCA: Principal component analysis
qRT‒PCR: Quantitative–reverse transcription polymerase chain reaction
ROI: Region of interest
ROS: Reactive oxygen species
SD: Standard deviation
SNP: Single nucleotide polymorphisms

## References

[1] Report AA. 2020 Alzheimer’s disease facts and figures. Alzheimers Dement. 2020;16:391–460.

[2] Scheltens P, De Strooper B, Kivipelto M, Holstege H, Chetelat G, Teunissen CE, Cummings J, van der Flier WM. Alzheimer’s disease. Lancet. 2021;397:1577–90. 33667416 10.1016/S0140-6736(20)32205-4 PMC8354300

[3] Hollingworth P, Harold D, Sims R, Gerrish A, Lambert JC, Carrasquillo MM, Abraham R, Hamshere ML, Pahwa JS, Moskvina V, et al. Common variants at ABCA7, MS4A6A/MS4A4E, EPHA1, CD33 and CD2AP are associated with Alzheimer’s disease. Nat Genet. 2011;43:429–35. 21460840 10.1038/ng.803 PMC3084173

[4] Kaminski WE, Orso E, Diederich W, Klucken J, Drobnik W, Schmitz G. Identification of a novel human sterol-sensitive ATP-binding cassette transporter (ABCA7). Biochem Biophys Res Commun. 2000;273:532–8. 10873640 10.1006/bbrc.2000.2954

[5] Dib S, Pahnke J, Gosselet F. Role of ABCA7 in Human Health and in Alzheimer’s Disease. Int J Mol Sci. 2021;22(9):4603. 10.3390/ijms22094603 10.3390/ijms22094603 PMC8124837 33925691

[6] Lyssenko NN, Pratico D. ABCA7 and the altered lipidostasis hypothesis of Alzheimer’s disease. Alzheimers Dement. 2021;17:164–74. 33336544 10.1002/alz.12220 PMC7986801

[7] Aikawa T, Holm ML, Kanekiyo T. ABCA7 and Pathogenic Pathways of Alzheimer’s Disease. Brain Sci. 2018;8(2):27. 10.3390/brainsci8020027 10.3390/brainsci8020027 PMC5836046 29401741

[8] Jehle AW, Gardai SJ, Li S, Linsel-Nitschke P, Morimoto K, Janssen WJ, Vandivier RW, Wang N, Greenberg S, Dale BM, et al. ATP-binding cassette transporter A7 enhances phagocytosis of apoptotic cells and associated ERK signaling in macrophages. J Cell Biol. 2006;174:547–56. 16908670 10.1083/jcb.200601030 PMC2064260

[9] Tanaka N, Abe-Dohmae S, Iwamoto N, Fitzgerald ML, Yokoyama S. Helical apolipoproteins of high-density lipoprotein enhance phagocytosis by stabilizing ATP-binding cassette transporter A7. J Lipid Res. 2010;51:2591–9. 20495215 10.1194/jlr.M006049 PMC2918442

[10] Aikawa T, Ren Y, Yamazaki Y, Tachibana M, Johnson MR, Anderson CT, Martens YA, Holm ML, Asmann YW, Saito T, et al. ABCA7 haplodeficiency disturbs microglial immune responses in the mouse brain. Proc Natl Acad Sci U S A. 2019;116:23790–6. 31690660 10.1073/pnas.1908529116 PMC6876254

[11] Nowyhed HN, Chandra S, Kiosses W, Marcovecchio P, Andary F, Zhao M, Fitzgerald ML, Kronenberg M, Hedrick CC. ATP binding Cassette Transporter ABCA7 regulates NKT Cell Development and function by Controlling CD1d expression and lipid raft content. Sci Rep. 2017;7:40273. 28091533 10.1038/srep40273 PMC5238393

[12] Singh D. Astrocytic and microglial cells as the modulators of neuroinflammation in Alzheimer’s disease. J Neuroinflammation. 2022;19:206. 35978311 10.1186/s12974-022-02565-0 PMC9382837

[13] Rueda-Carrasco J, Martin-Bermejo MJ, Pereyra G, Mateo MI, Borroto A, Brosseron F, Kummer MP, Schwartz S, Lopez-Atalaya JP, Alarcon B, et al. SFRP1 modulates astrocyte-to-microglia crosstalk in acute and chronic neuroinflammation. EMBO Rep. 2021;22:e51696. 34569685 10.15252/embr.202051696 PMC8567217

[14] Bhusal A, Afridi R, Lee WH, Suk K. Bidirectional Communication between Microglia and astrocytes in Neuroinflammation. Curr Neuropharmacol. 2023;21:2020–9. 36453496 10.2174/1570159X21666221129121715 PMC10556371

[15] Frohlich C, Paarmann K, Steffen J, Stenzel J, Krohn M, Heinze HJ, Pahnke J. Genomic background-related activation of microglia and reduced beta-amyloidosis in a mouse model of Alzheimer’s disease. Eur J Microbiol Immunol (Bp). 2013;3:21–7. 23814667 10.1556/EuJMI.3.2013.1.3 PMC3694725

[16] Matejuk A, Ransohoff RM. Crosstalk between astrocytes and microglia: an overview. Front Immunol. 2020;11:1416. 32765501 10.3389/fimmu.2020.01416 PMC7378357

[17] Wu Y, Eisel ULM. Microglia-Astrocyte communication in Alzheimer’s Disease. J Alzheimers Dis. 2023;95:785–803. 37638434 10.3233/JAD-230199 PMC10578295

[18] Villa M, Wu J, Hansen S, Pahnke J. Emerging role of ABC Transporters in Glia Cells in Health and diseases of the Central Nervous System. Cells. 2024;13(9):740. 10.3390/cells13090740 10.3390/cells13090740 PMC11083179 38727275

[19] Van Zeller M, Dias D, Sebastiao AM, Valente CA. NLRP3 inflammasome: a starring role in amyloid-beta- and tau-driven pathological events in Alzheimer’s Disease. J Alzheimers Dis. 2021;83:939–61. 34366341 10.3233/JAD-210268 PMC8543248

[20] Liu Y, Dai Y, Li Q, Chen C, Chen H, Song Y, Hua F, Zhang Z. Beta-amyloid activates NLRP3 inflammasome via TLR4 in mouse microglia. Neurosci Lett. 2020;736:135279. 32726591 10.1016/j.neulet.2020.135279

[21] Jung ES, Suh K, Han J, Kim H, Kang HS, Choi WS, Mook-Jung I. Amyloid-beta activates NLRP3 inflammasomes by affecting microglial immunometabolism through the Syk-AMPK pathway. Aging Cell. 2022;21:e13623. 35474599 10.1111/acel.13623 PMC9124305

[22] Ibrahim WW, Skalicka-Wozniak K, Budzynska B, El Sayed NS. NLRP3 inflammasome inhibition and M1-to-M2 microglial polarization shifting via scoparone-inhibited TLR4 axis in ovariectomy/D-galactose Alzheimer’s disease rat model. Int Immunopharmacol. 2023;119:110239. 37137264 10.1016/j.intimp.2023.110239

[23] Halle A, Hornung V, Petzold GC, Stewart CR, Monks BG, Reinheckel T, Fitzgerald KA, Latz E, Moore KJ, Golenbock DT. The NALP3 inflammasome is involved in the innate immune response to amyloid-beta. Nat Immunol. 2008;9:857–65. 18604209 10.1038/ni.1636 PMC3101478

[24] Song L, Pei L, Yao S, Wu Y, Shang Y. NLRP3 inflammasome in neurological diseases, from functions to therapies. Front Cell Neurosci. 2017;11:63. 28337127 10.3389/fncel.2017.00063 PMC5343070

[25] Tschopp J, Schroder K. NLRP3 inflammasome activation: the convergence of multiple signalling pathways on ROS production? Nat Rev Immunol. 2010;10:210–5. 20168318 10.1038/nri2725

[26] Heneka MT, Kummer MP, Stutz A, Delekate A, Schwartz S, Vieira-Saecker A, Griep A, Axt D, Remus A, Tzeng TC, et al. NLRP3 is activated in Alzheimer’s disease and contributes to pathology in APP/PS1 mice. Nature. 2013;493:674–8. 23254930 10.1038/nature11729 PMC3812809

[27] van Kralingen C, Kho DT, Costa J, Angel CE, Graham ES. Exposure to inflammatory cytokines IL-1beta and TNFalpha induces compromise and death of astrocytes; implications for chronic neuroinflammation. PLoS ONE. 2013;8:e84269. 24367648 10.1371/journal.pone.0084269 PMC3868583

[28] Hyvarinen T, Hagman S, Ristola M, Sukki L, Veijula K, Kreutzer J, Kallio P, Narkilahti S. Co-stimulation with IL-1beta and TNF-alpha induces an inflammatory reactive astrocyte phenotype with neurosupportive characteristics in a human pluripotent stem cell model system. Sci Rep. 2019;9:16944. 31729450 10.1038/s41598-019-53414-9 PMC6858358

[29] Radde R, Bolmont T, Kaeser SA, Coomaraswamy J, Lindau D, Stoltze L, Calhoun ME, Jaggi F, Wolburg H, Gengler S, et al. Abeta42-driven cerebral amyloidosis in transgenic mice reveals early and robust pathology. EMBO Rep. 2006;7:940–6. 16906128 10.1038/sj.embor.7400784 PMC1559665

[30] Batth TS, Tollenaere MX, Ruther P, Gonzalez-Franquesa A, Prabhakar BS, Bekker-Jensen S, Deshmukh AS, Olsen JV. Protein aggregation capture on Microparticles enables Multipurpose Proteomics Sample Preparation. Mol Cell Proteom. 2019;18:1027–35. 10.1074/mcp.TIR118.001270 PMC6495262 30833379

[31] Skowronek P, Meier F. High-throughput Mass Spectrometry-based proteomics with dia-PASEF. Methods Mol Biol. 2022;2456:15–27. 35612732 10.1007/978-1-0716-2124-0_2

[32] Tyanova S, Temu T, Cox J. The MaxQuant computational platform for mass spectrometry-based shotgun proteomics. Nat Protoc. 2016;11:2301–19. 27809316 10.1038/nprot.2016.136

[33] Bascunana P, Brackhan M, Mohle L, Wu J, Bruning T, Eiriz I, Jansone B, Pahnke J. Time- and Sex-Dependent Effects of Fingolimod Treatment in a Mouse Model of Alzheimer’s Disease. Biomolecules. 2023;13(2):331. 10.3390/biom13020331 10.3390/biom13020331 PMC9953119 36830699

[34] Mohle L, Stefan K, Bascunana P, Brackhan M, Bruning T, Eiriz I, El Menuawy A, van Genderen S, Santos-Garcia I, Gorska AM et al. ABC Transporter C1 Prevents Dimethyl Fumarate from Targeting Alzheimer’s Disease. Biology. 2023;12(7):932. 10.3390/biology12070932 10.3390/biology12070932 PMC10376064 37508364

[35] Brackhan M, Calza G, Lundgren K, Bascunana P, Bruning T, Soliymani R, Kumar R, Abelein A, Baumann M, Lalowski M, Pahnke J. Isotope-labeled amyloid-beta does not transmit to the brain in a prion-like manner after peripheral administration. EMBO Rep. 2022;23:e54405. 35620875 10.15252/embr.202154405 PMC9253763

[36] Mohle L, Brackhan M, Bascunana P, Pahnke J. Dimethyl fumarate does not mitigate cognitive decline and beta-amyloidosis in female APPPS1 mice. Brain Res. 2021;1768:147579. 34233173 10.1016/j.brainres.2021.147579

[37] Farfara D, Sooliman M, Avrahami L, Royal TG, Amram S, Rozenstein-Tsalkovich L, Trudler D, Blanga-Kanfi S, Eldar-Finkelman H, Pahnke J, et al. Physiological expression of mutated TAU impaired astrocyte activity and exacerbates beta-amyloid pathology in 5xFAD mice. J Neuroinflammation. 2023;20:174. 37496076 10.1186/s12974-023-02823-9 PMC10369740

[38] Steffen J, Krohn M, Schwitlick C, Bruning T, Paarmann K, Pietrzik CU, Biverstal H, Jansone B, Langer O, Pahnke J. Expression of endogenous mouse APP modulates beta-amyloid deposition in hAPP-transgenic mice. Acta Neuropathol Commun. 2017;5:49. 28637503 10.1186/s40478-017-0448-2 PMC5480119

[39] Scheffler K, Stenzel J, Krohn M, Lange C, Hofrichter J, Schumacher T, Bruning T, Plath AS, Walker L, Pahnke J. Determination of spatial and temporal distribution of microglia by 230nm-high-resolution, high-throughput automated analysis reveals different amyloid plaque populations in an APP/PS1 mouse model of Alzheimer’s disease. Curr Alzheimer Res. 2011;8:781–8. 21244350 10.2174/156720511797633179 PMC3117051

[40] Mohle L, Bascunana P, Brackhan M, Pahnke J. Development of deep learning models for microglia analyses in brain tissue using DeePathology STUDIO. J Neurosci Methods. 2021;364:109371. 34592173 10.1016/j.jneumeth.2021.109371

[41] Bascunana P, Brackhan M, Pahnke J. Machine learning-supported analyses improve quantitative histological assessments of amyloid-beta deposits and activated Microglia. J Alzheimers Dis. 2021;79:597–605. 33337377 10.3233/JAD-201120 PMC7902967

[42] Guler BE, Krzysko J, Wolfrum U. Isolation and culturing of primary mouse astrocytes for the analysis of focal adhesion dynamics. STAR Protoc. 2021;2:100954. 34917973 10.1016/j.xpro.2021.100954 PMC8669101

[43] Tyanova S, Temu T, Sinitcyn P, Carlson A, Hein MY, Geiger T, Mann M, Cox J. The Perseus computational platform for comprehensive analysis of (prote)omics data. Nat Methods. 2016;13:731–40. 27348712 10.1038/nmeth.3901

[44] Goedhart J, Luijsterburg MS. VolcaNoseR is a web app for creating, exploring, labeling and sharing volcano plots. Sci Rep. 2020;10:20560. 33239692 10.1038/s41598-020-76603-3 PMC7689420

[45] Kuleshov MV, Jones MR, Rouillard AD, Fernandez NF, Duan Q, Wang Z, Koplev S, Jenkins SL, Jagodnik KM, Lachmann A, et al. Enrichr: a comprehensive gene set enrichment analysis web server 2016 update. Nucleic Acids Res. 2016;44:W90–97. 27141961 10.1093/nar/gkw377 PMC4987924

[46] Xie Z, Bailey A, Kuleshov MV, Clarke DJB, Evangelista JE, Jenkins SL, Lachmann A, Wojciechowicz ML, Kropiwnicki E, Jagodnik KM, et al. Gene Set Knowledge Discovery with Enrichr. Curr Protoc. 2021;1:e90. 33780170 10.1002/cpz1.90 PMC8152575

[47] Chen EY, Tan CM, Kou Y, Duan Q, Wang Z, Meirelles GV, Clark NR. Ma’ayan A: Enrichr: interactive and collaborative HTML5 gene list enrichment analysis tool. BMC Bioinformatics. 2013;14:128. 23586463 10.1186/1471-2105-14-128 PMC3637064

[48] Li X, Li C, Zhang W, Wang Y, Qian P, Huang H. Inflammation and aging: signaling pathways and intervention therapies. Signal Transduct Target Ther. 2023;8:239. 37291105 10.1038/s41392-023-01502-8 PMC10248351

[49] Lasry A, Ben-Neriah Y. Senescence-associated inflammatory responses: aging and cancer perspectives. Trends Immunol. 2015;36:217–28. 25801910 10.1016/j.it.2015.02.009

[50] Rachmian N, Medina S, Cherqui U, Akiva H, Deitch D, Edilbi D, Croese T, Salame TM, Ramos JMP, Cahalon L, et al. Identification of senescent, TREM2-expressing microglia in aging and Alzheimer’s disease model mouse brain. Nat Neurosci. 2024;27:1116–24. 38637622 10.1038/s41593-024-01620-8

[51] Broz P, Dixit VM. Inflammasomes: mechanism of assembly, regulation and signalling. Nat Rev Immunol. 2016;16:407–20. 27291964 10.1038/nri.2016.58

[52] Dash PK, Gorantla S, Poluektova L, Hasan M, Waight E, Zhang C, Markovic M, Edagwa B, Machhi J, Olson KE, et al. Humanized mice for infectious and neurodegenerative disorders. Retrovirology. 2021;18:13. 34090462 10.1186/s12977-021-00557-1 PMC8179712

[53] Zhong MZ, Peng T, Duarte ML, Wang M, Cai D. Updates on mouse models of Alzheimer’s disease. Mol Neurodegener. 2024;19:23. 38462606 10.1186/s13024-024-00712-0 PMC10926682

[54] Baglietto-Vargas D, Forner S, Cai L, Martini AC, Trujillo-Estrada L, Swarup V, Nguyen MMT, Do Huynh K, Javonillo DI, Tran KM, et al. Generation of a humanized abeta expressing mouse demonstrating aspects of Alzheimer’s disease-like pathology. Nat Commun. 2021;12:2421. 33893290 10.1038/s41467-021-22624-z PMC8065162

[55] Krohn M, Wanek T, Menet MC, Noack A, Decleves X, Langer O, Loscher W, Pahnke J. Humanization of the blood-brain barrier transporter ABCB1 in mice disrupts genomic locus - lessons from three unsuccessful approaches. Eur J Microbiol Immunol (Bp). 2018;8:78–86. 30345087 10.1556/1886.2018.00008 PMC6186017

[56] Krohn M, Zoufal V, Mairinger S, Wanek T, Paarmann K, Bruning T, Eiriz I, Brackhan M, Langer O, Pahnke J. Generation and characterization of an Abcc1 Humanized Mouse Model (hABCC1(flx/flx)) with knockout capability. Mol Pharmacol. 2019;96:138–47. 31189668 10.1124/mol.119.115824

[57] Iqbal J, Suarez MD, Yadav PK, Walsh MT, Li Y, Wu Y, Huang Z, James AW, Escobar V, Mokbe A, et al. ATP-binding cassette protein ABCA7 deficiency impairs sphingomyelin synthesis, cognitive discrimination, and synaptic plasticity in the entorhinal cortex. J Biol Chem. 2022;298:102411. 36007616 10.1016/j.jbc.2022.102411 PMC9513280

[58] Lamartiniere Y, Boucau MC, Dehouck L, Krohn M, Pahnke J, Candela P, Gosselet F, Fenart L. ABCA7 downregulation modifies Cellular cholesterol homeostasis and decreases amyloid-beta peptide efflux in an in vitro model of the blood-brain barrier. J Alzheimers Dis. 2018;64:1195–211. 30010117 10.3233/JAD-170883

[59] Kim WS, Li H, Ruberu K, Chan S, Elliott DA, Low JK, Cheng D, Karl T, Garner B. Deletion of Abca7 increases cerebral amyloid-beta accumulation in the J20 mouse model of Alzheimer’s disease. J Neurosci. 2013;33:4387–94. 23467355 10.1523/JNEUROSCI.4165-12.2013 PMC6704948

[60] Krohn M, Lange C, Hofrichter J, Scheffler K, Stenzel J, Steffen J, Schumacher T, Bruning T, Plath AS, Alfen F, et al. Cerebral amyloid-beta proteostasis is regulated by the membrane transport protein ABCC1 in mice. J Clin Invest. 2011;121:3924–31. 21881209 10.1172/JCI57867 PMC3195473

[61] Capolupo A, Cassiano C, Casapullo A, Andreotti G, Cubellis MV, Riccio A, Riccio R, Monti MC. Identification of Trombospondin-1 as a Novel Amelogenin interactor by functional proteomics. Front Chem. 2017;5:74. 29057222 10.3389/fchem.2017.00074 PMC5635807

[62] De Roeck A, Van Broeckhoven C, Sleegers K. The role of ABCA7 in Alzheimer’s disease: evidence from genomics, transcriptomics and methylomics. Acta Neuropathol. 2019;138:201–20. 30903345 10.1007/s00401-019-01994-1 PMC6660495

[63] Yvan-Charvet L, Wang N, Tall AR. Role of HDL, ABCA1, and ABCG1 transporters in cholesterol efflux and immune responses. Arterioscler Thromb Vasc Biol. 2010;30:139–43. 19797709 10.1161/ATVBAHA.108.179283 PMC2812788

[64] Butler CA, Mendoza Arvilla A, Milinkeviciute G, Da Cunha C, Kawauchi S, Rezaie N, Liang HY, Javonillo D, Thach A, Wang S, et al. The Abca7(V1613M) variant reduces Abeta generation, plaque load, and neuronal damage. Alzheimers Dement. 2024;20:4914–34. 38506634 10.1002/alz.13783 PMC11247689

[65] Patani R, Hardingham GE, Liddelow SA. Functional roles of reactive astrocytes in neuroinflammation and neurodegeneration. Nat Rev Neurol. 2023;19:395–409. 37308616 10.1038/s41582-023-00822-1

[66] Linnerbauer M, Wheeler MA, Quintana FJ. Astrocyte crosstalk in CNS inflammation. Neuron. 2020;108:608–22. 32898475 10.1016/j.neuron.2020.08.012 PMC7704785

[67] Sofroniew MV. Molecular dissection of reactive astrogliosis and glial scar formation. Trends Neurosci. 2009;32:638–47. 19782411 10.1016/j.tins.2009.08.002 PMC2787735

[68] Wiener JP, Desire S, Garliyev V, Lyssenko Iii N, Pratico D, Lyssenko NN. Down-regulation of ABCA7 in human microglia, astrocyte and THP-1 cell lines by cholesterol depletion, IL-1beta and TNFalpha, or PMA. Cells. 2023;12(17):2143. 10.3390/cells12172143 10.3390/cells12172143 PMC10486366 37681876

[69] Duchateau L, Wawrzyniak N, Sleegers K. The ABC’s of Alzheimer risk gene ABCA7. Alzheimers Dement. 2024;20:3629–48. 38556850 10.1002/alz.13805 PMC11095487

[70] Kim J, Yoo ID, Lim J, Moon JS. Pathological phenotypes of astrocytes in Alzheimer’s disease. Exp Mol Med. 2024;56:95–9. 38172603 10.1038/s12276-023-01148-0 PMC10834520

[71] Bai H, Zhang Q. Activation of NLRP3 inflammasome and onset of Alzheimer’s Disease. Front Immunol. 2021;12:701282. 34381452 10.3389/fimmu.2021.701282 PMC8350495

[72] Stewart CR, Stuart LM, Wilkinson K, van Gils JM, Deng J, Halle A, Rayner KJ, Boyer L, Zhong R, Frazier WA, et al. CD36 ligands promote sterile inflammation through assembly of a toll-like receptor 4 and 6 heterodimer. Nat Immunol. 2010;11:155–61. 20037584 10.1038/ni.1836 PMC2809046

[73] Pelegrin P. P2X7 receptor and the NLRP3 inflammasome: Partners in crime. Biochem Pharmacol. 2021;187:114385. 33359010 10.1016/j.bcp.2020.114385

[74] Li S, Fang Y, Zhang Y, Song M, Zhang X, Ding X, Yao H, Chen M, Sun Y, Ding J, et al. Microglial NLRP3 inflammasome activates neurotoxic astrocytes in depression-like mice. Cell Rep. 2022;41:111532. 36288697 10.1016/j.celrep.2022.111532

[75] Choi SS, Lee HJ, Lim I, Satoh J, Kim SU. Human astrocytes: secretome profiles of cytokines and chemokines. PLoS ONE. 2014;9:e92325. 24691121 10.1371/journal.pone.0092325 PMC3972155

[76] Liddelow SA, Guttenplan KA, Clarke LE, Bennett FC, Bohlen CJ, Schirmer L, Bennett ML, Munch AE, Chung WS, Peterson TC, et al. Neurotoxic reactive astrocytes are induced by activated microglia. Nature. 2017;541:481–7. 28099414 10.1038/nature21029 PMC5404890

[77] Hou B, Zhang Y, Liang P, He Y, Peng B, Liu W, Han S, Yin J, He X. Inhibition of the NLRP3-inflammasome prevents cognitive deficits in experimental autoimmune encephalomyelitis mice via the alteration of astrocyte phenotype. Cell Death Dis. 2020;11:377. 32415059 10.1038/s41419-020-2565-2 PMC7229224

[78] Podcasy JL, Epperson CN. Considering sex and gender in Alzheimer disease and other dementias. Dialogues Clin Neurosci. 2016;18:437–46. 28179815 10.31887/DCNS.2016.18.4/cepperson PMC5286729

[79] Fu Y, He Y, Phan K, Pickford R, Kim YB, Dzamko N, Halliday GM, Kim WS. Sex-specific lipid dysregulation in the Abca7 knockout mouse brain. Brain Commun. 2022;4:fcac120. 35620166 10.1093/braincomms/fcac120 PMC9127619

[80] Iwamoto N, Abe-Dohmae S, Sato R, Yokoyama S. ABCA7 expression is regulated by cellular cholesterol through the SREBP2 pathway and associated with phagocytosis. J Lipid Res. 2006;47:1915–27. 16788211 10.1194/jlr.M600127-JLR200

[81] Stein B, Yang MX. Repression of the interleukin-6 promoter by estrogen receptor is mediated by NF-kappa B and C/EBP beta. Mol Cell Biol. 1995;15:4971–9. 7651415 10.1128/mcb.15.9.4971 PMC230744

[82] El Sabeh R, Bonnet M, Le Corf K, Lang K, Kfoury A, Badran B, Hussein N, Virard F, Treilleux I, Le Romancer M, et al. A gender-dependent Molecular switch of inflammation via MyD88/Estrogen Receptor-Alpha Interaction. J Inflamm Res. 2021;14:2149–56. 34045885 10.2147/JIR.S306805 PMC8149287

