# Supplementary material for: The ABC transporter A7 modulates neuroinflammation via NLRP3 inflammasome in Alzheimer’s disease mice
Source: Alzheimers Res Ther. 2025 Jan 27;17:30. doi: 10.1186/s13195-025-01673-2 (PMC11773842; doi:10.1186/s13195-025-01673-2)
Supplement: Supplementary file 1 — Supplementary Material 1: Table S1. Primer sequences for genotyping of the Abca7 / ABCA7 locus. Table S2. Comparison of DNA and protein sequences of murine, human and the in-frame inserted construct. Start codon for translation in the mouse gene is located in exon 2. Fig. S1. Agarose gel showing different PCR fragment sizes for each genotype. Fig. S2. Isolation of CD11b+ microglial cells from 100-day-old APPPS1 and APPPS1-hA7ko mice. Fig. S3. NLRP3 inflammasome changes induced by ABCA7 total knockout versus conditional Cx3cr1-knockout in 200-day-old APPPS1 mice. Fig. S4. Correlations between soluble Aβ42 levels (TBS fraction) and mRNA expression of NLRP3 inflammasome activators P2rx7 (A) and Cd36 (B). Fig. S5. LPS stimulation induces NLRP3 inflammasome expression and pro/anti-inflammatorycytokines release in APPPS1-hA7ko microglia [file 13195_2025_1673_MOESM1_ESM.pdf]

SUPPLEMENTARY MATERIAL

DOI: 10.1186/s13195-025-01673-2

The ABC transporter A7 modulates neuroinflammation via NLRP3 inflammasome in Alzheimer's disease mice

Irene Santos-García [1], Pablo Bascuñana [1], Mirjam Brackhan [1], María Villa [1], Ivan Eiriz [1], Thomas Brüning [1], Jens Pahnke [1,2,3,4,*]

[1] Translational Neurodegeneration Research and Neuropathology Lab, Department of Clinical Medicine (KlinMed), Medical Faculty, University of Oslo (UiO) and Section of Neuropathology Research, Department of Pathology (PAT), Clinics for Laboratory Medicine (KLM), Oslo University Hospital (OUS), Sognsvannsveien 20, NO-0372 Oslo, Norway, (www.pahnkelab.eu)

[2] Institute of Nutritional Medicine (INUM) / Lübeck Institute of Dermatology (LIED), University of Lübeck (UzL) and University Medical Center Schleswig-Holstein (UKSH), Ratzeburger Allee 160, DE-23538 Lübeck, Germany

[3] Department of Neuromedicine and Neuroscience, The Faculty of Medicine and Life Sciences, University of Latvia (LU), Jelgavas iela 3, LV-1004 Rīga, Latvia

[4] Department of Neurobiology, School of Neurobiology, Biochemistry and Biophysics, The Georg S. Wise Faculty of Life Sciences, Tel Aviv University (TAU), Ramat Aviv, IL-6997801, Israel

Content:

Table S1. Primer sequences for genotyping of the *Abca7* / *ABCA7* locus

Table S2. Comparison of DNA and protein sequences of murine, human and the in-frame inserted construct. Start codon for translation in the mouse gene is located in exon 2.

Figure S1. Agarose gel showing different PCR fragment sizes for each genotype

Figure S2. Isolation of CD11b^{+} microglial cells from 100-day-old APPPS1 and APPPS1-hA7^{ko} mice.

Figure S3. NLRP3 inflammasome changes induced by *ABCA7* total knockout versus conditional *Cx3cr1*-knockout in 200-day-old APPPS1 mice.

Figure S4. Correlations between soluble Aβ42 levels (TBS fraction) and mRNA expression of NLRP3 inflammasome activators *P2rx7* (A) and *Cd36* (B).

Figure S5. LPS stimulation induces NLRP3 inflammasome expression and pro/anti-inflammatorycytokines release in APPPS1-hA7^{ko} microglia.

Table S1. Primer sequences for genotyping of the *Abca7 / ABCA7* locus.

Gene	Primer sequence
***hABCA7* forward**	5’ – AGAATCTTGCCTACAAATCCAGG – 3’
***hABCA7* reverse**	5’ – GGTCTTTGTATCCTGTGCCA – 3’
***hABCA7*_Cre**	5’ – ATTTACAGCCCCAGGTCTCTAC – 3’

Table S2. Comparison of DNA and protein sequences of murine, human and the in-frame inserted construct. Start codon for translation in the mouse gene is located in exon 2.

Locus	Sequence
mouse Abca7 cDNA	ABCA7_Mouse_cDNA_sequence.txt
**mouse Abca7 protein**	ABCA7_Mouse_Protein_sequence.txt
***Human ABCA7* cDNA**	ABCA7_Chimeric_cDNA_sequence.txt
**Human ABCA7 protein**	Translation of ABCA7_Chimeric_cDNA_sequence.txt
Abca7$^{\mathbf{tm1.1(\mathit{ABCA7})Pahnk}}$	Abca7tm1.1(ABCA7)Pahnk_locus.txt
**Abca7**$^{\mathbf{tm1.2(ABCA7ko)Pahnk}}$	Abca7tm1.2Pahnk_KO_locus.txt

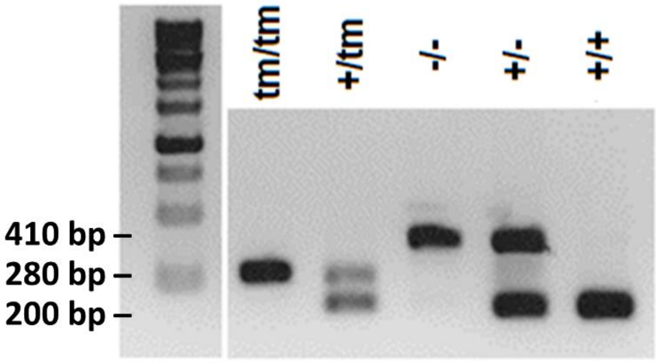

Figure S1. Agarose gel showing different PCR fragment sizes for each genotype: homologous recombinant *ABCA7* in the *Abca7* locus (tm/tm; 280 bp), heterozygous recombinant *Abca7/ABCA7* (+/tm; 200 and 280 bp), humanized *ABCA7* knockout (-/-; 410 bp), haplodeficient *Abca7*/- (+/-; 200 and 410 bp) and wild-type *Abca7/Abca7* (+/+; 200 bp).

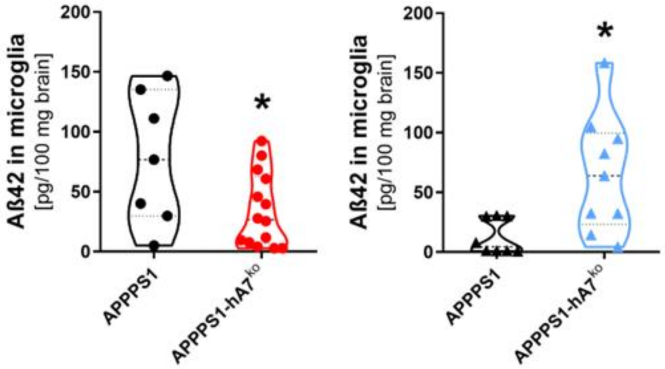

Figure S2. Isolation of CD11b$^+$ microglial cells from 100-day-old APPPS1 and APPPS1-hA7^{ko} mice. Quantitation of Aβ_{42} levels in isolated CD11b$^+$ microglia from 100-day-old females (●) and males (▲). The data are presented as the means ± SDs (n = 9-10/experimental group); significance was determined using Student's t test (*$p \leq 0.05$).

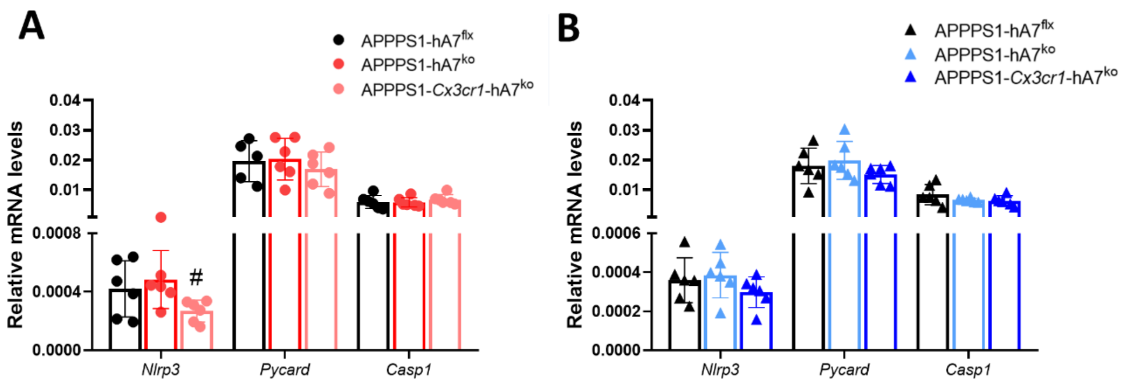

Figure S3. NLRP3 inflammasome changes induced by ABCA7 total knockout *versus* conditional *Cx3cr1* knockout in 200-days-old APPPS1 mice. mRNA expression levels quantified by qPCR of genes involved in NLRP3 inflammasome oligomerization (A, B). Data are presented as mean ± SD (n = 6 / experimental group); significance was calculated using a one-way ANOVA test followed by Tukey *post-hoc* test (# p ≤ 0.05 vs. APPPS1-hA7^{ko}).

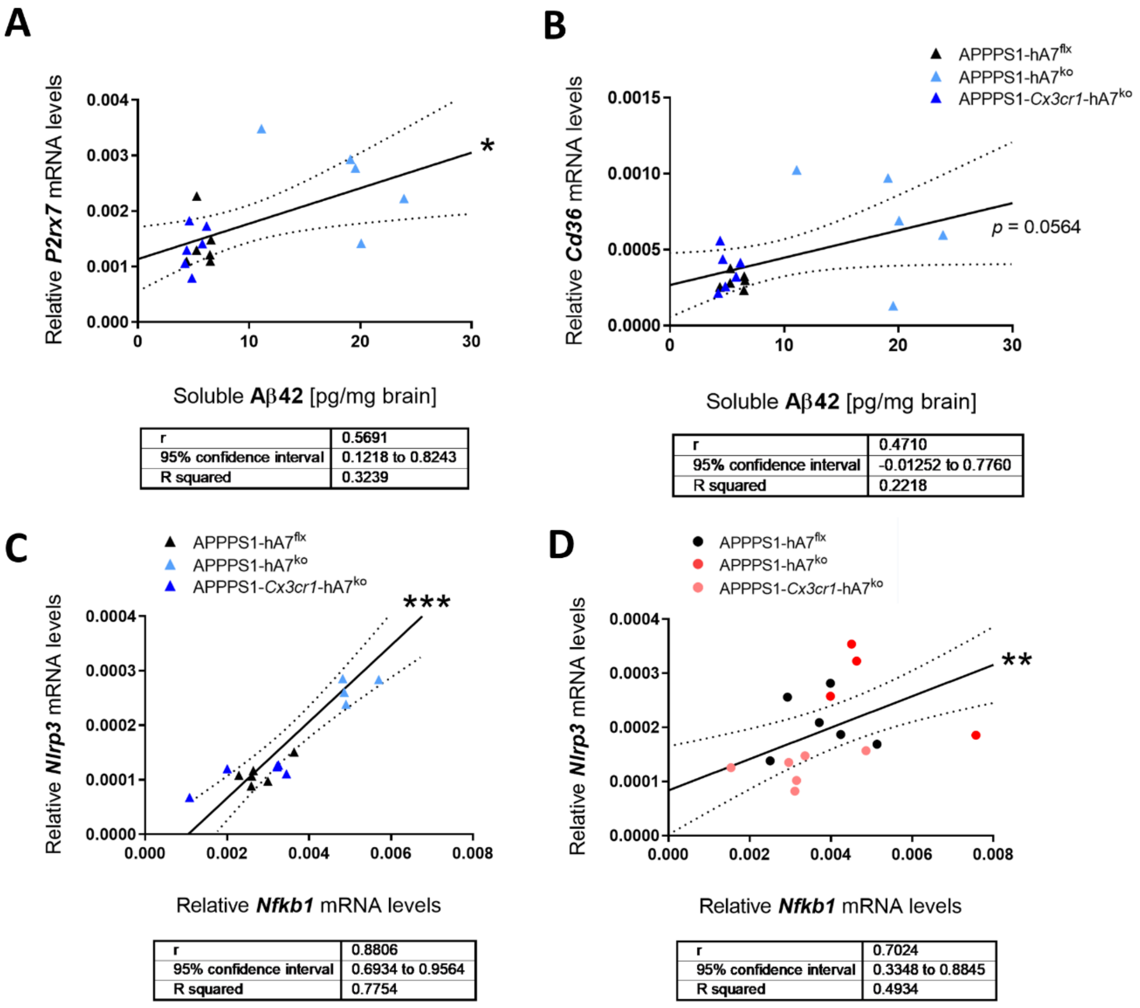

A

r	0.5691
95% confidence interval	0.1218 to 0.8243
R squared	0.3239

B

r	0.4710
95% confidence interval	-0.01252 to 0.7760
R squared	0.2218

C

r	0.8806
95% confidence interval	0.6934 to 0.9564
R squared	0.7754

D

r	0.7024
95% confidence interval	0.3348 to 0.8845
R squared	0.4934

Figure S4. Correlations between soluble Aβ42 levels (TBS fraction) and mRNA expression of NLRP3 inflammasome activators *P2rx7* (A) and *Cd36* (B). Correlation between *Nlrp3* and *Nfkb1* mRNA expression in males (C) and females (D). Data are presented as mean ± SD (n = 5-6 / experimental group); significance was calculated using regression line (* p ≤ 0.05, ** p ≤ 0.01, *** p ≤ 0.001).

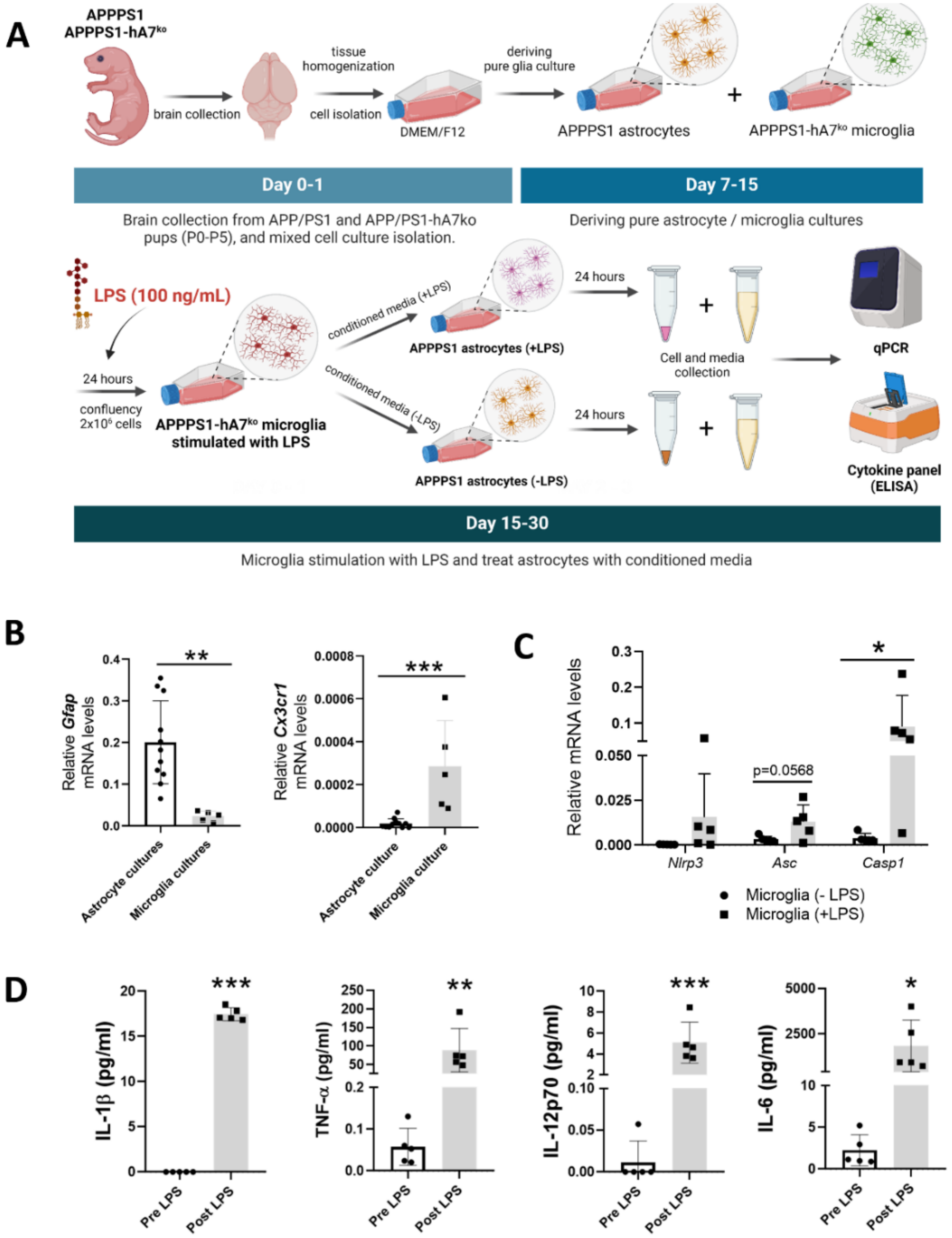

Figure S5. LPS stimulation induces NLRP3 inflammasome expression and pro/anti-inflammatory cytokines release in APPPS1-hA7^{ko} microglia. Scheme of the protocol used to perform conditioned primary glia cultures from APPPS1 and APPPS1-hA7^{ko} pups (created with BioRender.com) (A). Purity checking of astrocyte and microglia primary cell cultures by qPCR analysis of *Gfap* and *Cx3cr1* markers (B). mRNA expression levels quantified by qPCR of genes involved in NLRP3 inflammasome oligomerization on APPPS1-hA7^{ko} microglia cells before and after LPS induction for 24 hours (C). Levels of pro-inflammatory selected cytokines quantified by ELISA on APPPS1-hA7^{ko} microglia cells before and after LPS induction for 24 hours (D). Data are presented as mean ± SD (n = 5 independent cultures / experimental condition); significance was calculated using Student's t-test (* $p \leq 0.05$, ** $p \leq 0.01$, *** $p \leq 0.001$).
